# A fluorescent ESIPT-based benzimidazole platform for the ratiometric two-photon imaging of ONOO^–^*in vitro* and *ex vivo*†
†Electronic supplementary information (ESI) available. See DOI: 10.1039/d0sc02347g


**DOI:** 10.1039/d0sc02347g

**Published:** 2020-06-16

**Authors:** Maria L. Odyniec, Sang-Jun Park, Jordan E. Gardiner, Emily C. Webb, Adam C. Sedgwick, Juyoung Yoon, Steven D. Bull, Hwan Myung Kim, Tony D. James

**Affiliations:** a Department of Chemistry , University of Bath , BA2 7AY , UK . Email: T.D.James@bath.ac.uk ; Email: S.D.Bull@bath.ac.uk; b Department of Chemistry , Ajou University , 16499 , Suwon , Korea . Email: kimhm@ajou.ac.kr; c Department of Chemistry , University of Texas at Austin , 105 E, 24th Street , A5300 , Austin , USA; d Department of Chemistry and Nano Science , Ewha Womans University , Seoul 120-750 , Korea

## Abstract

In this work, we have developed an ESIPT-based benzimidazole platform (**MO-E1** and **MO-E2**) for the two-photon cell imaging of ONOO^–^ and a potential ONOO^–^-activated theranostic scaffold (**MO-E3**). Each benzimidazole platform, **MO-E1–3**, were shown to rapidly detect ONOO^–^ at micromolar concentrations (LoD = 0.28 μM, 6.53 μM and 0.81 μM respectively). The potential theranostic **MO-E3** was shown to release the parent fluorophore and drug indomethacin in the presence of ONOO^–^ but unfortunately did not perform well *in vitro* due to low solubility. Despite this, the parent scaffold **MO-E2** demonstrated its effectiveness as a two-photon imaging tool for the ratiometric detection of endogenous ONOO^–^ in RAW264.7 macrophages and rat hippocampus tissue. These results demonstrate the utility of this ESIPT benzimidazole-based platform for theranostic development and bioimaging applications.

## Abstract 2

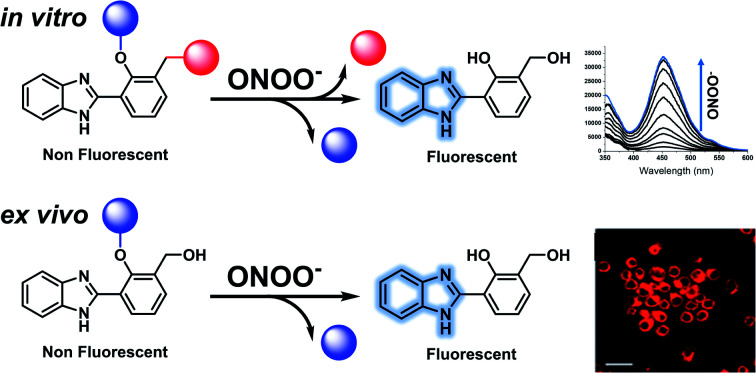
In this work, we have developed an ESIPT benzimidazole-based platform for the two-photon cell imaging of ONOO^–^ and a potential ONOO^–^-activated theranostic scaffold.

## Introduction

Fluorescent probes using bespoke chemical architectures are rationally designed to elicit a fluorescent response after reacting with a target molecule. Peroxynitrite (ONOO^–^) is a reactive nitrogen species (RNS) of interest. Low levels of ONOO^–^ are constituently produced under basal conditions, which are tolerated through thiol-dependent antioxidant detoxification pathways.^1^ However, high levels of ONOO^–^ are implicated in the pathological and physiological processes of multiple oxidative stress-related diseases, including neurodegenerative and inflammatory diseases.^2^ ONOO^–^ is a strong oxidant with a short half-life which can react with various biomolecules such as DNA and proteins, causing major oxidative injury.^3^ Due to this high reactivity and short lifetime, accurate detection of ONOO^–^ remains a challenging task.

One successfully implemented approach for the detection of ONOO^–^ uses boronate-based fluorescent probes.^4^,^5^ Previously, such probes have been used to detect hydrogen peroxide (H_2_O_2_). However, given the reaction rates of boronate esters with ONOO^–^ are 10^6^ times faster than H_2_O_2_, probes for the former have recently been developed.^6^ A variety of probes to detect ONOO^–^*via* different mechanisms are reported and are discussed elegantly in reviews by Shen *et al.* and Chan *et al.*^7^,^8^ Perhaps one of the most useful non-boronate probes for monitoring ONOO^–^ is the reversible near-IR cyanine dye modulated by a PET processes through selenium oxidation; designed by the Han group. A reversible signal is particularly useful for continuous monitoring real time changes in the levels of a biological analyte.^9^

Excited state intramolecular proton transfer (ESIPT) probes are attractive systems for developing reaction-based fluorescence systems since they have excellent photophysical properties, including good photostability and large Stokes shifts.^10^ In addition ratiometric responses can also be observed. A dual-emission ratiometric response is desirable as it can provide direct information about the concentration of a target analyte using internal calibration, which is not possible with a single emission system.^10^ ESIPT probes often incorporate; 2-(2′-hydroxyphenyl)benzimidazole (HBI), 2-(2′-hydroxyphenyl)benzoxazole (HBO) or 2-(2′-hydroxyphenyl)benzothiazole (HBT) in the framework.

**Fig. 1:**
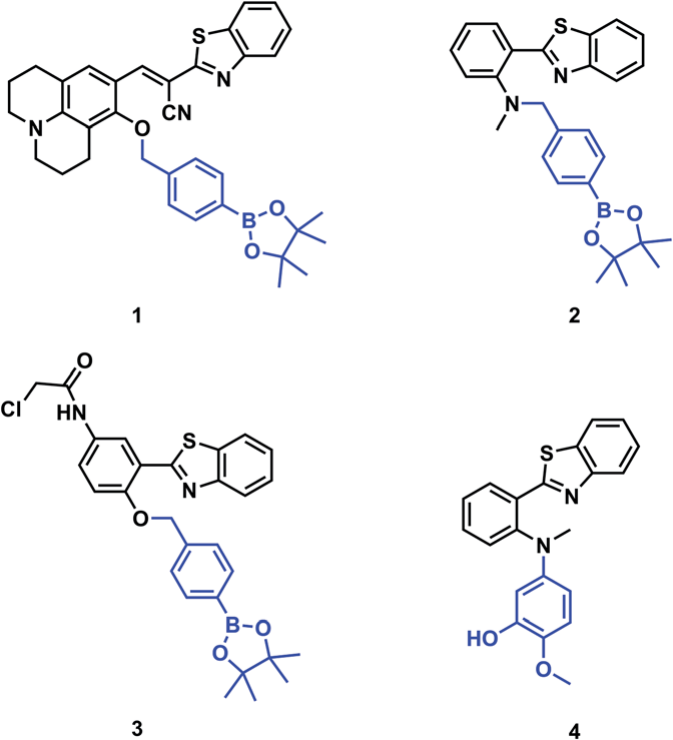
Structures of previously reported ONOO^–^ activatable probes.

Four fluorescent probes (**1–4**) for ONOO^–^ detection are illustrated in Fig. 1. Each of these probes use an HBT core. The first reported example, probe **1** designed by Kim *et al.*, used a benzothiazolyl iminocoumarin scaffold and a boronate ester activating group to enable endogenous imaging of ONOO^–^ in J774A.1 macrophages.^11^ Probes **2** and **3** designed by the James group incorporated the boronate ester onto a more simple benzothiazole scaffold for detection of ONOO^–^*in vitro*. Moreover, probe **3** was targeted to the endoplasmic reticulum (ER).^5^,^12^ Following a different mechanism of activation, probe **4** designed by Li *et al.* was used for visualisation of ONOO^–^ in neurovascular ischemia progression in the brain of a live mouse.^13^ As such, benzothiazoles have been shown to be good fluorescent probes for ONOO^–^ detection, with high selectivity, good cell permeability and potential to transport across the blood brain barrier.^13^

Due to the favourable properties of ESIPT based probes, we were interested in developing a potential ESIPT-based theranostic. Theranostics are the portmanteau of diagnostics and therapy. Typically, a theranostic will include an activating group, therapeutic group and a targeting group connected *via* a single linker.^12^,^14^

**Fig. 2:**
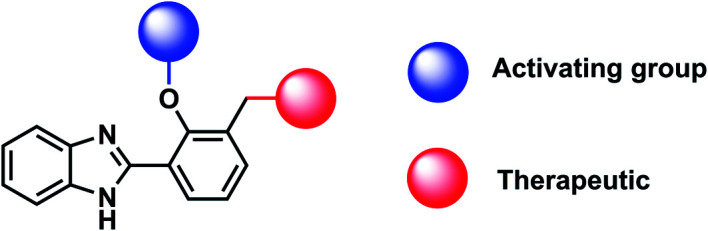
Design strategy towards ESIPT theranostics.

A common feature of theranostic linker building blocks are multiple primary alcohol functional groups, which need to be derivatised independently. This can lead to long tedious synthetic routes with multiple protection and deprotection steps. However, with our design strategy, we use the fluorophore as the ‘linker’ (Fig. 2).^15^,^16^ In this work, we have used HBI as the fluorophore ‘core’ and as the theranostic linker. Indomethacin was chosen as the therapeutic agent. Prominent HBI-based examples in the literature are probes which detect changes in pH. The Kim group developed a two-photon benzimidazole system to estimate acidic pH values in lysosomal compartments *in vitro* and *in vivo*.^17^ Liang and colleagues developed 3-benzimidazole-7-hydroxycoumarin which was able to detect two distinct pH ranges and monitor pH changes in the mitochondria of HeLa cells.^18^ From this work, we anticipated that our proposed benzimidazole system would provide a suitable platform for two-photon imaging of ONOO^–^ and provide the potential for theranostic applications (Fig. 2).

## Results and discussion

**Fig. 3:**
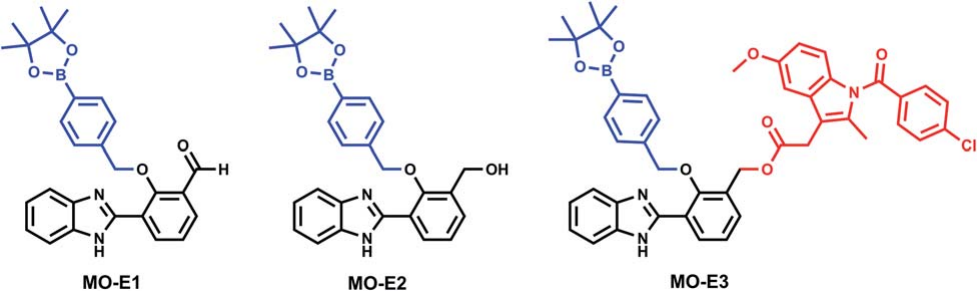
Structures of **MO-E1–3**.

In brief, for the synthesis of **MO-E1–3** (Fig. 3), commercially available HBI was subjected to duff reaction conditions (hexamethylenetetramine (HTMA) and trifluoroacetic acid (TFA)) to form intermediate 4 in a modest yield (35%); next, a mixture of 4,4-(4,4,5,5-tetramethyl-1,3,2-dioxaborolan-2-yl)benzyl bromide, and K_2_CO_3_ in dry DMF afforded **MO-E1** in 35% yield. The aldehyde functionality of **MO-E1** was reduced using NaBH_4_ to afford the corresponding alcohol **MO-E2** in 62% yield. Finally, **MO-E2** was conjugated to our chosen drug indomethacin using EDCI, in DMF to give **MO-E3** in 40% yield (Scheme S1, ESI†).

### Fluorescence analysis

The synthetic route to **MO-E3** provided a novel theranostic compound and two new fluorescent probes **MO-E1** and **MO-E2**. With these molecules in hand, spectroscopic analysis was performed. Data was collected in PBS buffer pH = 8.2 (52% w/w H_2_O : MeOH) at ambient temperature. Measurements were taken immediately after ONOO^–^ addition. The UV-Vis spectra of **MO-E1–3** (5 μM) were shown to have an absorption peak at 325 nm. The addition of ONOO^–^ led to an increase in absorption at 325 nm (Fig S2–S4, ESI†).

The mechanism of activation requires ONOO^–^ induced oxidation of an aryl boronate and subsequent elimination of the therapeutic. The boron atom of the boronate ester is sp^2^ hybridised and Lewis acidic. ONOO^–^ attacks the boron atom resulting in the formation of a peroxyborate intermediate. Subsequent aryl migration to the oxygen atom gives the boronate intermediate, which in the presence of water, undergoes quantitative hydrolysis to the corresponding phenol. Finally, a spontaneous 1,4-elimination results in release of the therapeutic leaving a reactive quinone methide, which on reaction with water forms the desired fluorescent adduct. The full mechanism can be found in the ESI (Fig S5, ESI†). Mass spectrometric analysis confirmed the formation of the expected fluorescent molecule (Fig S6–S9, ESI†).

**Fig. 4:**
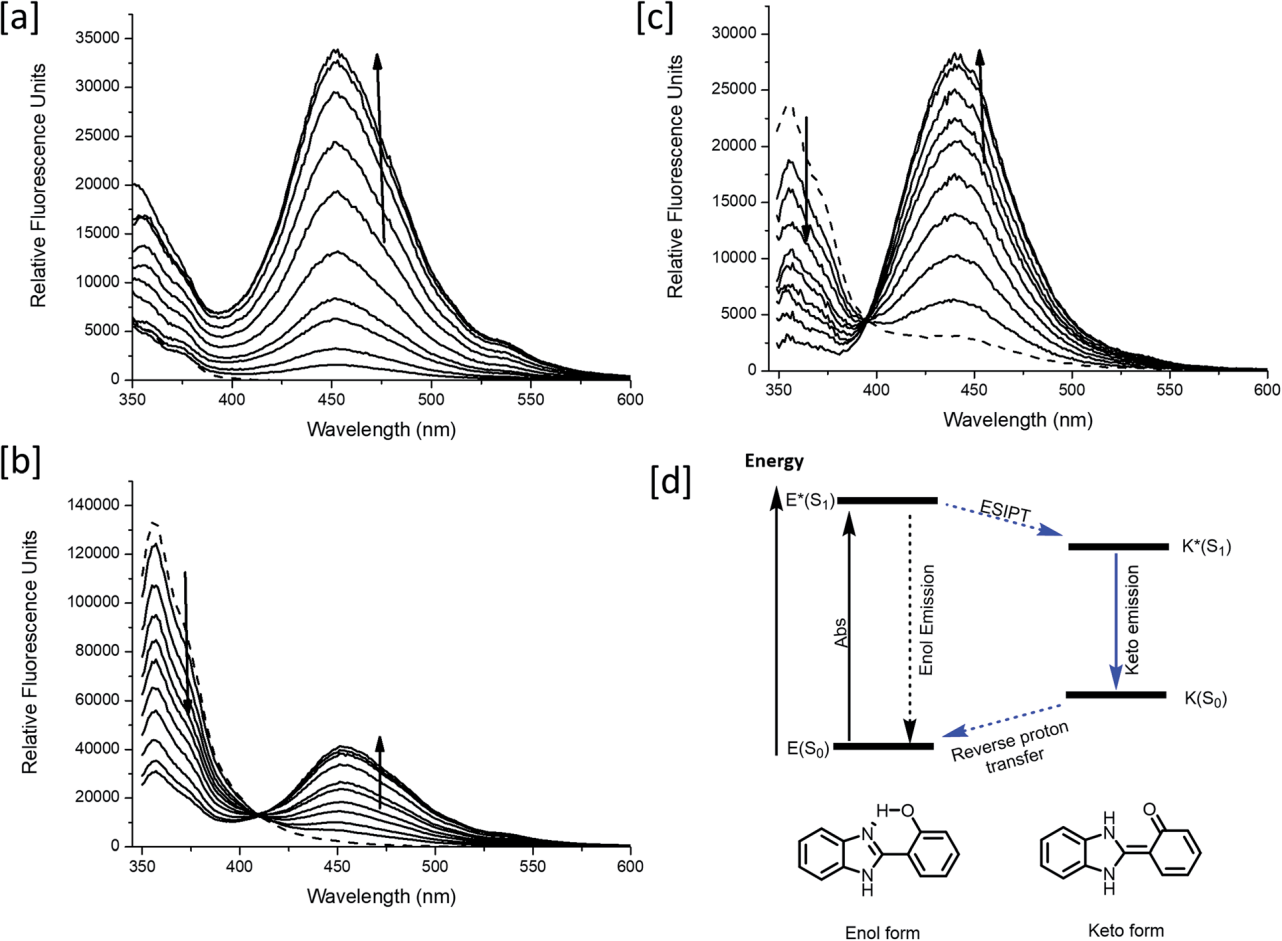
(a–c) Fluorescence spectra of **MO-E1–3** (5 μM) in the presence of ONOO^–^ (a) **MO-E3**: 0–30 μM, (b) **MO-E2**: 1–7 μM and (c) **MO-E1**: 1–5 μM. The dashed line represents sensor only and measurements were taken instantly after ONOO^–^ addition. All fluorescence measurements were measured in PBS buffer pH = 8.2 (52% w/w H_2_O : MeOH) at ambient temperature where *λ*_ex_ = 325 (bandwidth: 16) nm on a BMG Labtech CLARIOstar® plate reader. (d) ESIPT excited state diagram of HBI.

Following confirmation of the expected products, fluorescence studies were performed. Each probe (5 μM) was incubated with varying concentrations of ONOO^–^ and the fluorescence response was monitored (Fig. 4a–c). Initially, **MO-E3** was shown to be non-fluorescent. As the concentration of ONOO^–^ increased, the fluorescence response increased proportionally up to a plateau of 20 μM. The maximum fluorescence response was observed at *λ*_max_ = 450 nm (Fig. 4a). The limit of detection for ONOO^–^ is 0.28 μM (Fig. S13 and S14, ESI†). To ensure indomethacin was released from the system, LC-MS studies were performed using H_2_O_2_ as the reactive species. The data revealed the release of indomethacin, thus confirming **MO-E3** as a potential theranostic platform (Fig. S10–S12, ESI†). The cleavage of the ester was as expected from previous research in the James group.^19^

Unlike **MO-E3**, **MO-E1–2** elicited ratiometric responses in the presence of ONOO^–^ (Fig. 3b and c). Initially, both probes displayed a fluorescence emission intensity at 355 nm. This emission can be attributed to an ESIPT ‘enol’ form as the ESIPT process is blocked by the benzyl boronate ester. As the concentration of ONOO^–^ increased, (0–7 μM), a decrease in fluorescence emission at 355 nm with a concomitant increase at 450 nm was observed, characteristic of the ESIPT-based emission of HBI (Fig. 3e). The limit of detection for **MO-E1–2** were calculated to be 6.53 μM and 0.81 μM respectively (Fig S15–S18†). To date, the estimated steady state concentration of ONOO^–^ is in the nanomolar range.^20^–^22^ There are limited fluorescence methods for direct quantitation of ONOO^–^. Recently Wang and colleagues used a ratiometric BODIPY probe which measured average basal ONOO^–^ levels as 550–700 nM.^23^ Hence, **MO-E3** and **MO-E2** are well placed to monitor endogenous basal changes in ONOO^–^ as well as oxidative stress events where the concentration of ONOO^–^ is likely to increase significantly.

To test the probes ability to be activated by ONOO^–^ over other reactive oxygen species (ROS), selectivity studies were performed. **MO-E1–3** were tested against ROS (100 μM), ˙OH, O_2_˙^–^, ^1^O_2_, ClO^–^, ROO˙ and H_2_O_2_. As expected, the most significant fluorescence response was shown towards ONOO^–^. A 98-fold, 20-fold and 10-fold fluorescent enhancement was observed for **MO-E3**, **MO-E2** and **MO-E1** respectively at 450 nm (Fig. S19–S21†).

**Fig. 5:**
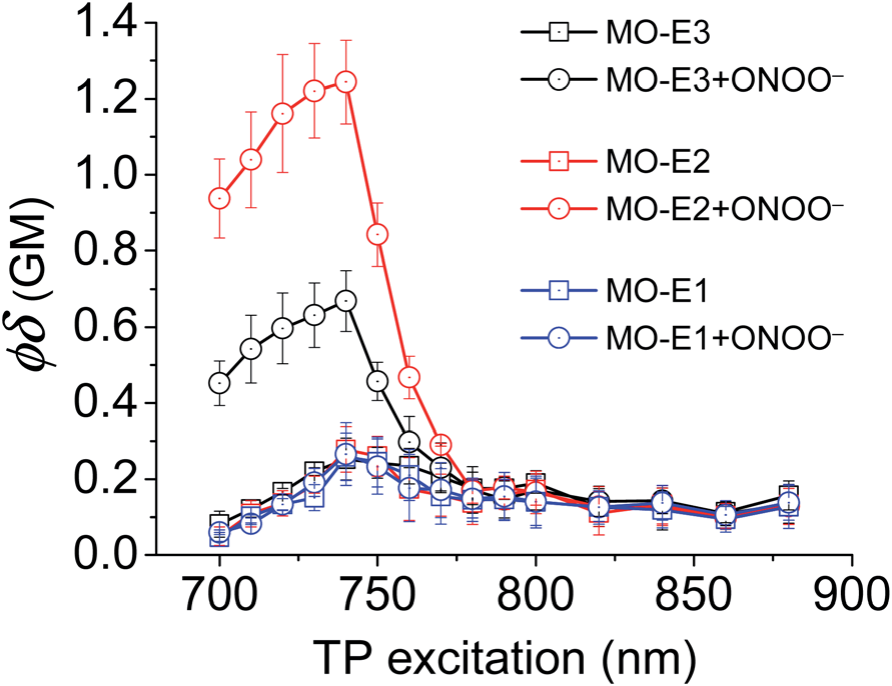
Two-photon action spectra of **MO-E** probes (5 μM) without and with ONOO^–^ (50 μM) in PBS buffer pH = 8.2 (52% w/w H_2_O : MeOH) at 25 °C.

Unfortunately, **MO-E1–3** were unsuitable for traditional cell imaging and confocal imaging experiments due to low excitation wavelengths (<360 nm). Therefore, to overcome this limitation each probe was evaluated towards two-photon microscopy (TPM). Unlike traditional fluorescence microscopy in which the excitation wavelength is shorter than the emission wavelength, in TPM the two photons excite at a longer wavelength than the resulting emitted light. This TPM strategy reduces short-wavelength induced phototoxicity and increases light penetration through living tissues with minimal light scattering.^24^ Before conducting TPM cell imaging experiments, two-photon action cross sections (TPA) of each ESIPT probe was measured (Fig. 5). TPA spectra for each probe before and after the addition of ONOO^–^ (50 μM) in PBS buffer (pH = 8.2, 52% w/w H_2_O : MeOH) were measured over a 700 nm–880 nm wavelength range. **MO-E3** and **MO-E2** reacted with ONOO^–^ and an increase in TPA was observed. All probes exhibited the highest TPA at 740 nm. However, **MO-E1** was unsuccessful for TPM imaging as only a minimal change was observed before and after the addition of ONOO^–^. The TPA values of each probe are shown in Table S1, ESI.†

TPM imaging of **MO-E3** was performed using RAW264.7 macrophages. RAW264.7 macrophages were incubated with **MO-E3** (5 μM) for 30 min and with a 740 nm excitation wavelength, **MO-E3** was successfully imaged (Fig S22†). However, intense spots on the outside of the cells were observed suggesting poor cell permeability/solubility for **MO-E3**. In addition, fluorescence intensities of intracellular **MO-E3** were almost unchanged, despite pre-treatment with 3-morpholinosydnonimine (SIN-1, 50 μM, 30 min), a ONOO^–^ donor, and exogenous ONOO^–^ (50 μM, 30 min). SIN-1 is the most widely used ONOO^–^ generator under physiological conditions. The sydnonimine ring of SIN-1 hydrolyses under aerobic aqueous conditions and releases nitric oxide and superoxide radicals, these two molecules combine to generate the ONOO^–^.^25^ Overall, we believe the limited solubility of **MO-E3** has resulted in its unsuccessful *in vitro* ONOO^–^ imaging. Nevertheless, **MO-E3** represents a proof of principle system which can allow for development and exploration into novel ratiometric ESIPT-based theranostics.

**Fig. 6:**
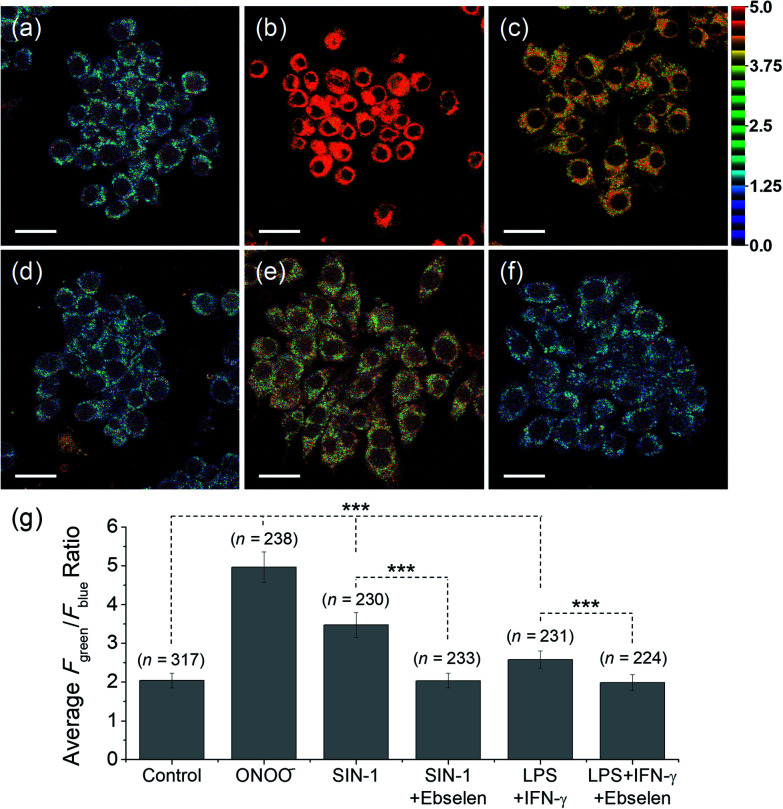
TPM ratiometric images of RAW264.7 macrophages labelled with **MO-E2** (5 μM) for 30 min. (a) Control image. (b–f) Cells were pre-treated with (b) exogenous ONOO^–^ (50 μM, 30 min), (c) SIN-1 (50 μM, 30 min), (d) SIN-1 and ebselen (50 μM, 30 min), (e) LPS (1 μg mL^–1^, 4 h) and IFN-γ (50 ng mL^–1^, 1 h), (f) LPS, IFN-γ, and ebselen (g) average ratio values in the corresponding TPM ratiometric images. Excitation wavelength was 740 nm and emission windows of each regions were 380–430 nm (*F*_blue_) and 470–520 nm (*F*_green_). Scale bars = 20 μm. Asterisks stand for the statistical significance (*p* < 0.001) and *n* is number of counted cells.

**Fig. 7:**
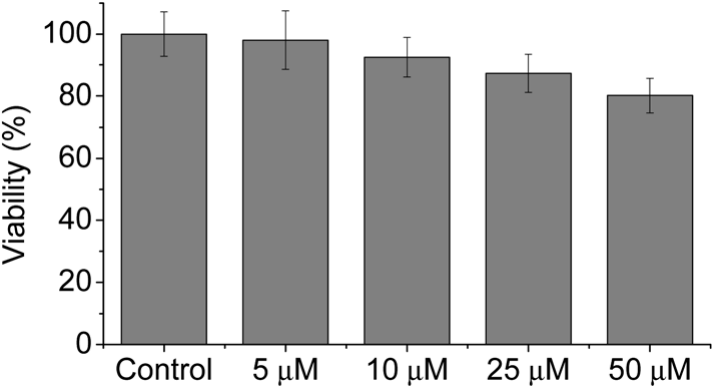
Cytotoxicity assays of **MO-E2** labelled RAW264.7 macrophages using CCK-8. Cells were incubated with 0–50 μM of **MO-E2** for 2 h.

**Fig. 8:**
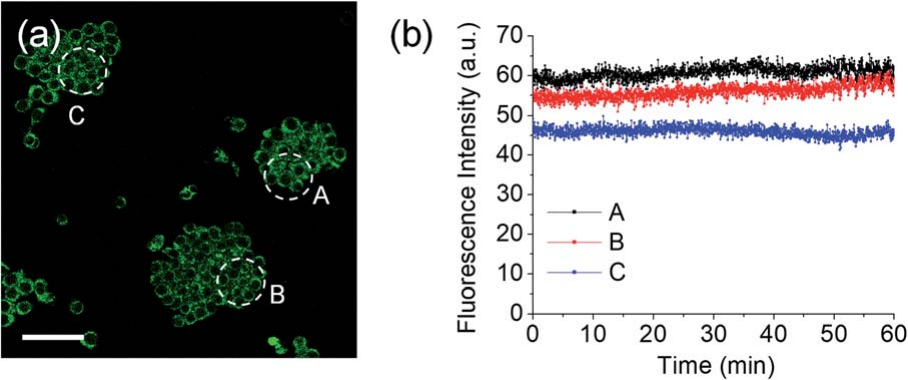
(a) TPM fluoresce images of RAW264.7 macrophages labelled with **MO-E2**. (b) The fluorescence intensities of areas A–C as a function of time from (a). The fluorescence intensity was obtained for 60 min at 2 s intervals. Excitation wavelength and detection windows were 740 nm and 380–600 nm, respectively.

Meanwhile, blue (380–430 nm, *F*_blue_) and green (470–520 nm, *F*_green_) regions were chosen as the detection windows for the ratiometric images (*F*_green_/*F*_blue_) of **MO-E2**. Unlike **MO-E3**, **MO-E2** (5 μM, 30 min) reacted with ONOO^–^ and displayed a large fluorescence change (Fig. 6). When pre-treated with exogenous ONOO^–^ (50 μM, 30 min) and SIN-1 (50 μM, 30 min), the average ratio values were significantly enhanced from 2.0 to 5.0 and 3.5, respectively (Fig. 6a–c). Additionally, cells were pre-treated with lipopolysaccharide (LPS, 1 μg mL^–1^, 4 h) and interferon gamma (INF-γ, 50 ng mL^–1^, 1 h), which are known to produce nitric oxide and superoxide to generate ONOO^–^ through induced iNOS expression and activated NADPH oxidase,^26^ and the average ratio value increased to 2.6 (Fig. 6e). Ebselen, an organoselenium compound, is a known ONOO^–^ scavenger that rapidly catalyses the reduction of ONOO^–^.^27^ When the stimulated macrophages were treated with ebselen (50 μM, 30 min), the average ratio values were unchanged and were at a similar level to those of the control (Fig. 6d and f). These results successfully demonstrate that **MO-E2** can be used to visualise ONOO^–^ in living cells. Furthermore, a CCK-8 assay demonstrated that **MO-E2** exhibited almost no cytotoxicity. When RAW264.7 cells were treated with **MO-E2** (50 μM) 80% of the cells survived (Fig. 7). The IC50 of **MO-E2** in RAW 264.7 cells for 24, 48, and 72 h were 164, 125 and 75 μM (Fig. S23†) respectively, confirming that **MO-E2** has negligible cytotoxicity under the imaging conditions. In addition, **MO-E2** has high photostability (Fig. 8). This was shown by incubating RAW264.7 cells with **MO-E2** followed by irradiation. The fluorescence intensities were obtained from 1800 signals with 2 s intervals for 1 h. Remarkably, the fluorescence emission intensity remained constant at 740 nm excitation wavelength over 1 h.

**Fig. 9:**
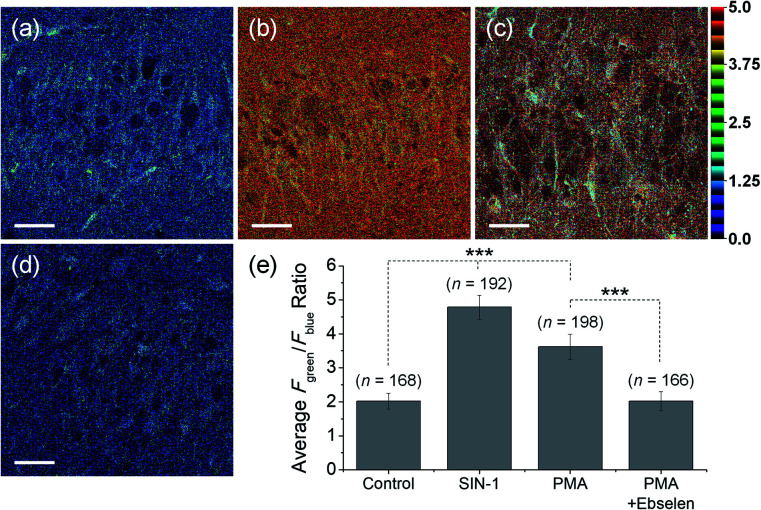
TPM ratiometric images of rat hippocampal slices stained with **MO-E2** (50 μM) for 1.5 h. (a) Control image. (b–d) Tissues were pre-treated with (b) SIN-1 (200 μM, 30 min), (c) PMA (20 μM, 30 min) (d) PMA and ebselen (200 μM, 30 min), (e) average ratio values in the corresponding TPM ratiometric images. Excitation wavelength was 740 nm and emission windows of each regions were 380–430 nm (*F*_blue_) and 470–520 nm (*F*_green_). Scale bars = 35 μm. Asterisks stand for the statistical significance (*p* < 0.001) and n is number of ROI from three samples for each imaging condition.

For the detection of ONOO^–^ in tissue, TPM is a powerful tool because of its lower excitation energy (>700 nm), deep tissue penetration depth, low photo damage and longer observation times. Due to the excellent photophysical properties of TPM and compatibility with **MO-E2**, we turned our attention towards the *ex vivo* imaging of ONOO^–^ using rat hippocampal tissue slices (Fig. 9). The average ratio values of tissues that had been stained with **MO-E2** (50 μM) for 1.5 h was 2.0, which was comparable to the macrophage control (Fig. 9a). When the hippocampal slice was pre-treated with SIN-1 (200 μM) for 30 min, the average ratio value was dramatically increased to 4.8 (Fig. 9b). When endogenous ROS was generated by treatment with PMA (20 μM, 30 min), the average ratio value was significantly increased to 3.6. While the addition ebselen suppressed the value similar-to that observed for the control (Fig. 9c and d). These results demonstrate the potential of **MO-E2** to be used as a two-photon ratiometric tool for the imaging of endogenous ONOO^–^ in living systems.

## Conclusions

With this work we demonstrate the utility of benzimidazoles for two-photon fluorescence imaging and as a reactive linker scaffold for the design of theranostic systems. Our approach used a simple synthetic route to prepare two-photon activatable probes, **MO-E1–3**. The probes were evaluated using two-photon microscopy. **MO-E1** did not produce large fluorescence changes on the addition of ONOO^–^ and as such was not evaluated further. Unfortunately, despite **MO-E3** illustrating the successful release of the fluorophore and therapeutic agent (mass spectrometry and LC-MS analysis), the system did not perform well *in vitro*. We believe this was due to the highly hydrophobic nature of indomethacin facilitating aggregation in the cell medium before the probe could enter cells. However, **MO-E2** excelled in visualising ONOO^–^ endogenously in RAW 264.7 macrophages and rat hippocampus tissue. The success of **MO-E2** in imaging ONOO^–^ using two-photon excitation indicates that a benzimidazole ‘linker’ is suitable for the construction of novel theranostic molecules incorporating more suitable drug candidates. Towards that end, we are currently exploring the development of such improved theranostic systems.

## Conflicts of interest

There are no conflicts to declare.

## Supplementary Material

Supplementary information
