# Supplementary material for: A fluorescent ESIPT-based benzimidazole platform for the ratiometric two-photon imaging of ONOO–in vitro and ex vivo
Source: Chem Sci. 2020 Jun 16;11(28):7329–34. doi: 10.1039/d0sc02347g (PMC7499849; doi:10.1039/d0sc02347g)
Supplement: Supplementary file 1 [file SC-011-D0SC02347G-s001.pdf]

Electronic Supplementary Material (ESI) for Chemical Science.
This journal is © The Royal Society of Chemistry 2020

Supplementary Information

A Fluorescent ESIPT Benzimidazole Platform for the Ratiometric Two-Photon Imaging of $ONOO^-$ *In Vitro* and *Ex Vivo*

Maria L. Odyniec,[a] Sang-Jun Park,[b] Jordan E. Gardiner,[a] Emily C. Webb,[a] Adam C. Sedgwick,[c] Juyoung Yoon,[d] Steven D. Bull,[a]* Hwan Myung Kim,*[b] Tony D. James.*[a]

[a]Department of Chemistry, University of Bath, BA2 7AY, UK. Email: T.D.James@bath.ac.uk S.D.Bull@bath.ac.uk

[b]Department of Chemistry, Ajou University, 16499, Suwon, Korea. Email: kimhm@ajou.ac.kr

[c]Department of Chemistry, University of Texas at Austin, 105 E, 24th Street, A5300, Austin, USA.

[d]Department of Chemistry and Nano Science, Ewha Womans University, Seoul 120-750, Korea.

Table of Contents

1. Experimental 2
2. NMR 7
3. Preparation of ROS/RNS 15
4. Materials and Methods 16
5. UV-VIS analysis 17
6. Mechanism of activation 19
7. Mass Spectrometry Analysis 20
8. Fluorescence Analysis 27
9. Cell studies 33

1. Experimental

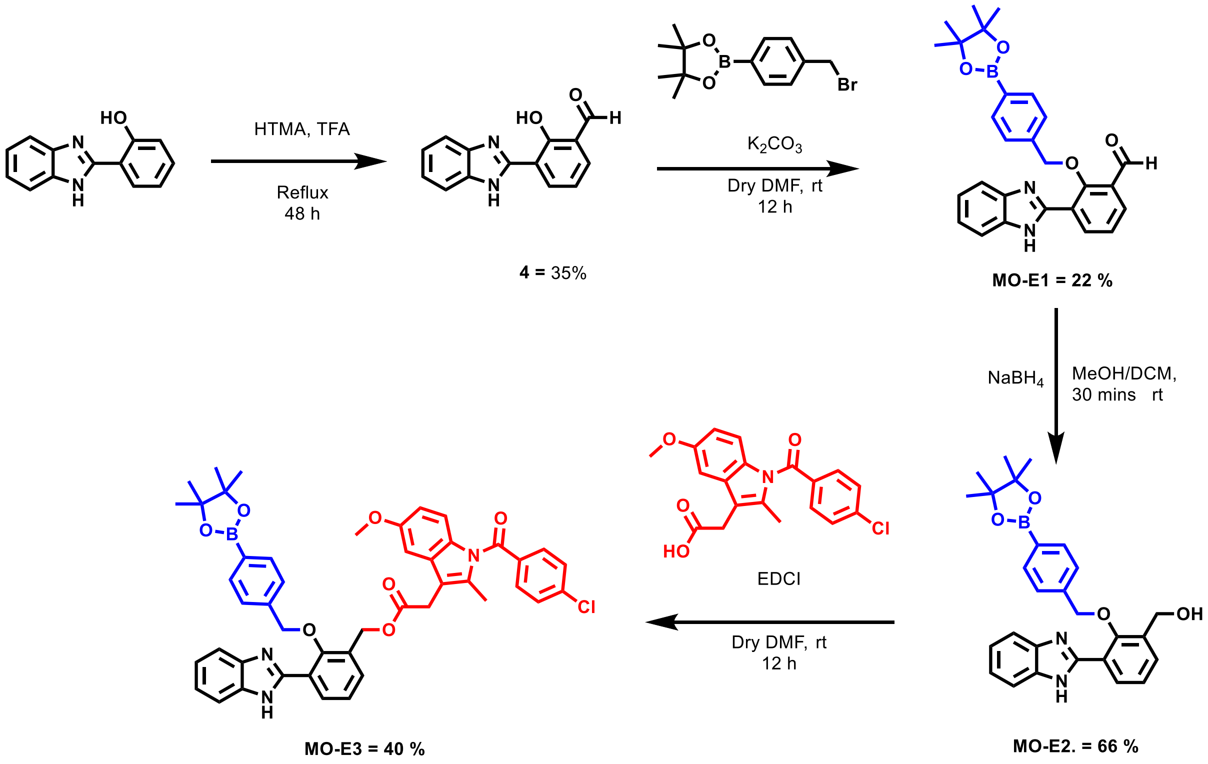

Figure S1 – Synthetic route to **MO-E1-3**

3-(1*H*-benzo[d]imidazol-2-yl)-2-hydroxybenzaldehyde (4)

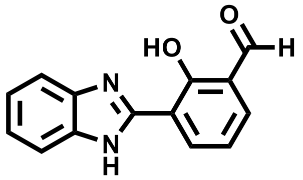

2-(1*H*-benzo[d]imidazol-2-yl)phenol (2.50 g, 11.9 mmol) and HMTA (3.33 g, 23.8 mmol) were added to trifluoroacetic acid (TFA - 42 mL) and refluxed for 48 h. After which, H_2O was added dropwise until a precipitate formed. The precipitate was extracted with warm EtOAc (50 mL) and washed with H_2O (2 x 30 mL), brine (50 mL), dried ($MgSO_4$), filtered and concentrated *in vacuo* to afford a bright yellow solid. The title compound was purified using column chromatography (97:3 DCM: MeOH) yielding a dark purple solid (1.00 g, 4.2 mmol, 35 %). ^{1}H NMR (500 MHz, DMSO-d_6) δ: 9.92 (s, 1H, -C*H*O), 8.66 (d, J = 1.8 Hz, 1H, Ar-*H*), 8.00 (dd, J = 8.5, 1.9 Hz, 1H, Ar-*H*), 7.75 (dd, J = 6.0, 3.1 Hz, 2H, Ar-*H*), 7.41 (dd, J = 5.9, 3.1 Hz, 2H, Ar-*H*), 7.28 (d, J = 8.5 Hz, 1H, Ar-*H*) ppm. ^{13}C NMR (126 MHz, DMSO-d_6) δ: 191.11, 163.18, 134.87, 130.07, 128.93, 124.74, 118.35, 115.08, 112.51, 79.61, 56.46 ppm. MP = 222-225 ·^{o}C. IR (thin film) ν max (cm^{-1}): 3404.90 (N-H), 3237.05 (O-H phenol), 2854.99 (C-H), 1666.75 (C=O). FTMS (p ESI): m/z calculated for $C_{14}H_{10}O_2N_2$ requires 238.0742 for $[M]^+$, found 238.0742.

3-(1*H*-benzo[d]imidazol-2-yl)-2-((4-(4,4,5,5-tetramethyl-1,3,2-dioxaborolan-2-yl)benzyl)oxy)benzaldehyde (MO-E1)

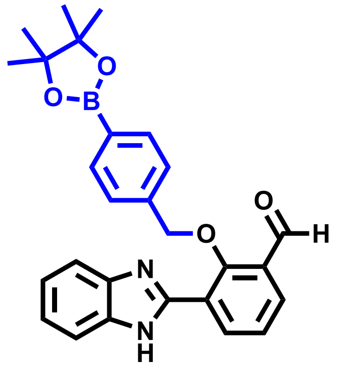

3-(1*H*-benzo[d]imidazol-2-yl)-2-hydroxybenzaldehyde (0.91 g, 3.81 mmol), 2-(4-(bromomethyl)phenyl)-4,4,5,5-tetramethyl-1,3,2-dioxaborolane (1.13, 3.81 mmol) and K_2CO_3 (0.63 g, 4.57 mmol) were added to dry DMF (6 mL) and stirred at rt for 12 h. After the allotted time, the reaction was diluted with EtOAc (30 mL), washed with H_2O (3 x 50 mL), brine (3 x 50 mL), dried ($MgSO_4$), filtered and concentrated *in vacuo*. The title compound was purified with column chromatography (20:80 EtOAc: pet ether) to afford the title compound as a white solid (0.35 g, 0.82 mmol, 22 %). ^{1}H NMR (500 MHz, DMSO-d_6) δ: 9.95 (s, 1H), 8.83 (d, J = 2.2 Hz, 1H, -C*H*O), 7.88 (dd, J = 8.6, 2.2 Hz, 1H, Ar-*H*), 7.69 (d, J = 7.7 Hz, 1H, Ar-*H*), 7.64 (t, J = 8.4 Hz, 3H, Ar-*H*), 7.50 (d, J = 7.8 Hz, 2H, Ar-*H*), 7.32 (d, J = 8.7 Hz, 1H, Ar-*H*), 7.28 – 7.19 (m, 3H, Ar-*H*), 5.66 (s, 2H, -O-CH_2-Ar), 1.25 (s, 12H, Bpin) ppm. ^{13}C NMR (126 MHz, DMSO-d_6) δ: 191.87, 160.10, 148.27, 140.21, 135.11, 132.64, 132.37, 130.31, 126.76, 123.01, 122.28, 119.98, 119.16, 114.78, 112.50, 84.12, 70.14, 25.09 ppm. MP = 199-201 ^{o}C. IR (thin film) ν max (cm^{-1}): 3432.41 (N-H), 2979.14 (C-H), 1695.98 (C=O). FTMS (p ESI): m/z calculated for $C_{27}H_{27}O_4N_2B$ requires 453.2100 for $[M+H]^+$, found 454.2223.

(3-(1*H*-benzo[d]imidazol-2-yl)-2-((4-(4,4,5,5-tetramethyl-1,3,2-dioxaborolan-2-yl)benzyl)oxy)phenyl)methanol (MO-E2)

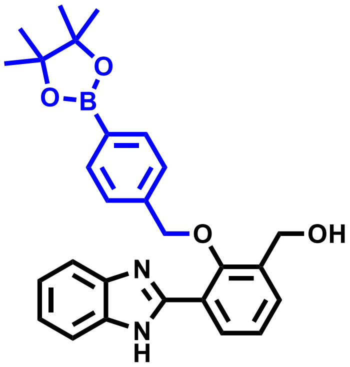

$NaBH_4$ (0.028 g, 0.74 mmol) in MeOH (1 mL) was added to a stirring solution of 3-(1*H*-benzo[d]imidazol-2-yl)-2-((4-(4,4,5,5-tetramethyl-1,3,2-dioxaborolan-2-yl)benzyl)oxy)benzaldehyde (0.32 g, 0. 74 mmol) in DCM (5 mL). The reaction was left to stir at rt for 30 mins, until completion by TLC. The mixture was diluted with DCM (20 mL), washed with H_2O (3 x 30 mL), brine (30 mL), dried ($MgSO_4$), filtered and concentrated *in vacuo* to afford the title compound as a white solid (0.21 g, 0.46 mmol, 62 %). No further purification was required. ^{1}H NMR (500 MHz, DMSO-d_6) δ: 8.26 (d, J = 2.2 Hz, 1H, Ar-*H*), 7.66 – 7.58 (m, 4H, Ar-*H*), 7.47 (d, J = 7.8 Hz, 2H, Ar-*H*), 7.25 (dd, J = 8.6, 2.3 Hz, 1H, Ar-*H*), 7.23 – 7.15 (m, 3H, Ar-*H*), 7.05 (d, J = 8.6 Hz, 1H, Ar-*H*), 5.52 (s, 2H, O-CH_2-Ar), 5.15 (q, J = 6.7, 6.3 Hz, 1H, O-*H*), 4.45 (d, J = 5.8 Hz, 2H, R-CH_2-OH), 1.25 (s, 12H, Bpin) ppm. ^{13}C NMR (126 MHz, DMSO-d_6) δ: 154.56, 149.58, 143.25, 141.07, 135.65, 135.19, 135.00, 129.60, 128.78, 126.74, 122.54, 121.97, 118.89, 113.96, 112.34, 84.09, 62.76, 25.10 ppm. MP = 239-240 ^{o}C. IR (thin film) ν max (cm^{-1}): 3404.68 (N-H), 3153.93 (O-H), 2934.16 (C-H). FTMS (p ESI): m/z calculated for $C_{27}H_{29}O_4N_2B$ requires 479.2113 for $[M]^+$, found 479.2117.

3-(1*H*-benzo[d]imidazol-2-yl)-2-((4-(4,4,5,5-tetramethyl-1,3,2-dioxaborolan-2-yl)benzyl)oxy)benzyl 2-(1-(4-chlorobenzoyl)-5-methoxy-2-methyl-1*H*-indol-3-yl)acetate (MO-E3)

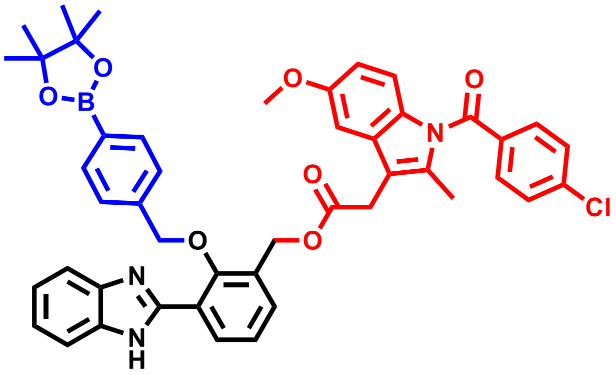

EDCI (50 µL) was added to a stirring solution of (3-(1*H*-benzo[d]imidazol-2-yl)-2-((4-(4,4,5,5-tetramethyl-1,3,2-dioxaborolan-2-yl)benzyl)oxy)phenyl)methanol (0.19 g, 0.41 mmol), indomethacin (0.15 g, 0.41 mmol) and DMAP (0.025 mmol, 0.2 mmol) in DCM (5 mL) and left for 12 h. The resulting solution was diluted with EtOAc (30 mL), washed with H_2O (3 x 30 mL), brine (3 x 30 mL), dried ($MgSO_4$) and concentrated *in vacuo*. The title compound was purified using column chromatography (50:50 % EtOAc: Petroleum Ether) to afford a pale yellow solid (0.13 g, 0.16 mmol, 40 %). ^{1}H NMR (500 MHz, $CDCl_3$) δ: 8.63 (s, 1H, Ar-*H*), 7.94 – 7.90 (m, 2H, Ar-*H*), 7.80 (d, *J* = 7.2 Hz, 1H, Ar-*H*), 7.63 – 7.60 (m, 3H, Ar-*H*), 7.52 (d, *J* = 7.9 Hz, 2H, Ar-*H*), 7.43 – 7.40 (m, 3H, Ar-*H*), 7.34 (d, *J* = 8.7 Hz, 2H, Ar-*H*), 7.06 (d, *J* = 8.6 Hz, 1H, Ar-*H*), 6.92 (d, *J* = 2.5 Hz, 1H, Ar-*H*), 6.86 (d, *J* = 9.0 Hz, 1H, Ar-*H*), 6.64 (dd, *J* = 9.0, 2.5 Hz, 1H, Ar-*H*), 5.33 (s, 2H, Ar-CH_2-O-R), 5.17 (s, 2H, Ar-O-CH_2-Ar), 3.75 (s, 3H ,R-O-CH_3), 3.71 (s, 2H, CO-CH_2-C-R), 2.36 (s, 3H, RN-C-CH_3), 1.38 (s, 12H, Bpin) ppm. ^{13}C NMR (126 MHz, $CDCl_3$) δ: 170.65, 168.24, 156.01, 155.88, 139.14, 138.51, 135.92, 135.56, 135.11, 135.02, 133.90, 131.12, 130.76, 130.56, 130.50, 129.02, 126.80, 126.03, 125.90, 114.90, 113.02, 112.47, 111.89, 101.07, 84.06, 76.98, 76.73, 75.00, 71.49, 66.11, 55.63, 30.39, 24.89, 24.85, 24.81 ppm. MP = 96-97 ^{o}C. IR (thin film) ν max (cm^{-1}): 3432.69 (N-H), 2932.19 (C-H), 1735.21, 1679.43 (C=O). FTMS (p ESI): m/z calculated for $C_{46}H_{43}O_7N_3BCl$ requires 794.2905for $[M]^+$, found 794.2919.

2. NMR

3-(1*H*-benzo[d]imidazol-2-yl)-2-hydroxybenzaldehyde (4) (500 MHz DMSO-D_6)

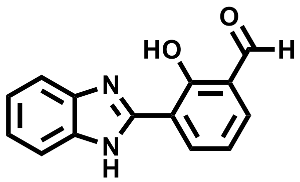

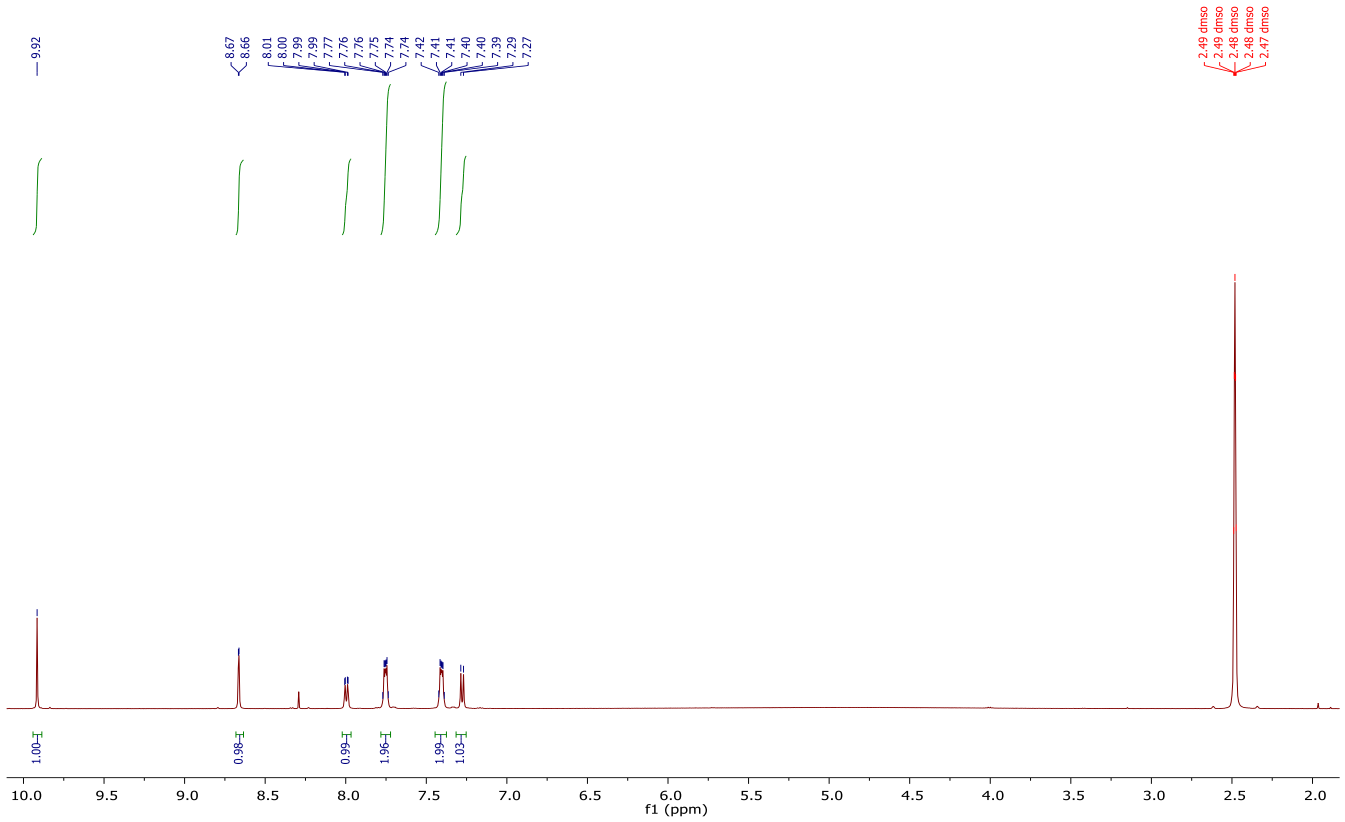

3-(1*H*-benzo[d]imidazol-2-yl)-2-hydroxybenzaldehyde (4) (126 MHz, DMSO-d_6)

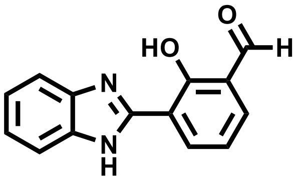

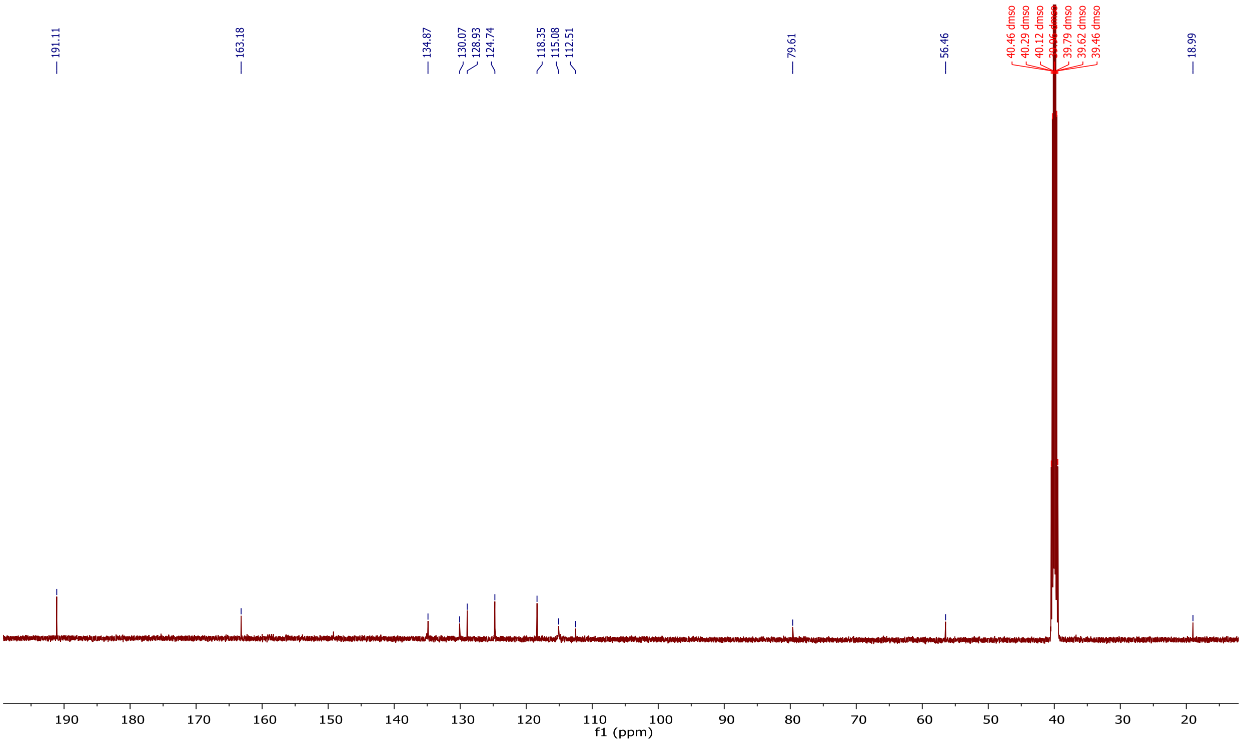

3-(1*H*-benzo[d]imidazol-2-yl)-2-((4-(4,4,5,5-tetramethyl-1,3,2-dioxaborolan-2-yl)benzyl)oxy)benzaldehyde (MO-E1) (500 MHz, DMSO-d_6)

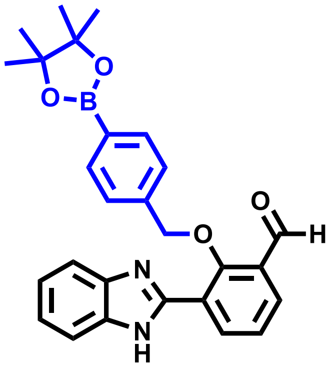

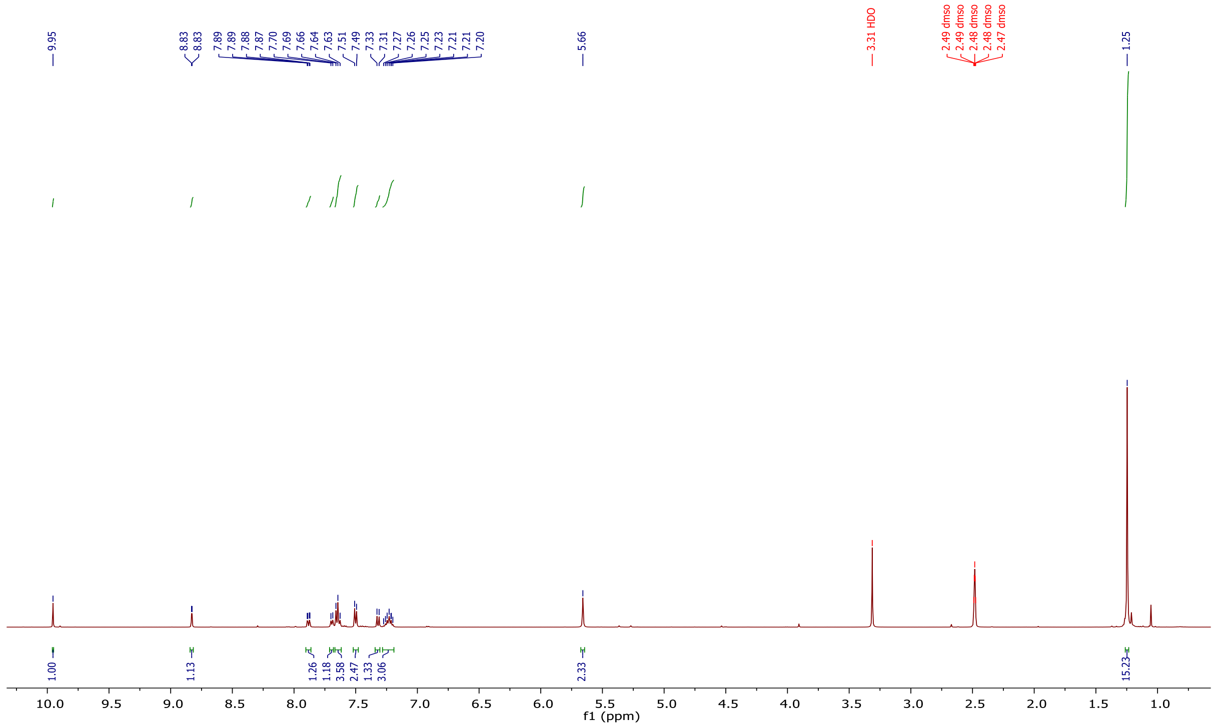

3-(1*H*-benzo[d]imidazol-2-yl)-2-((4-(4,4,5,5-tetramethyl-1,3,2-dioxaborolan-2-yl)benzyl)oxy)benzaldehyde (MO-E2) (126 MHz, DMSO-d_6)

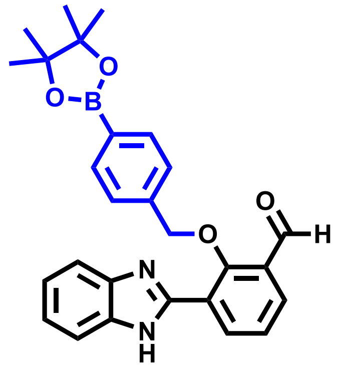

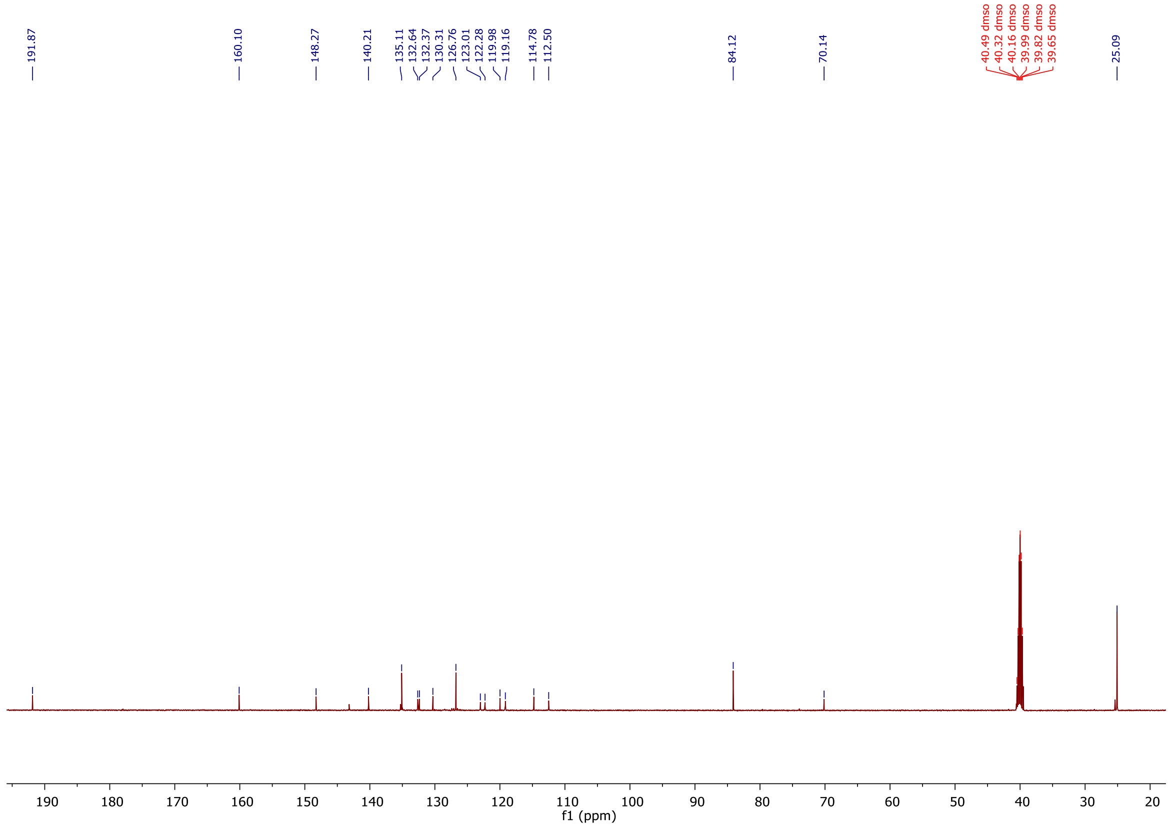

(3-(1*H*-benzo[d]imidazol-2-yl)-2-((4-(4,4,5,5-tetramethyl-1,3,2-dioxaborolan-2-yl)benzyl)oxy)phenyl)methanol (MO-E2) (500 MHz, DMSO-d_6)

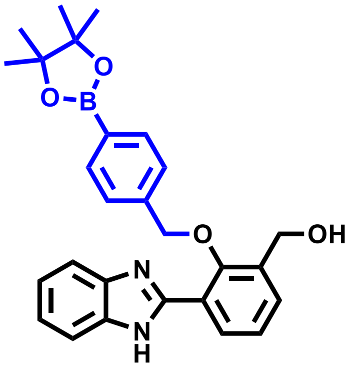

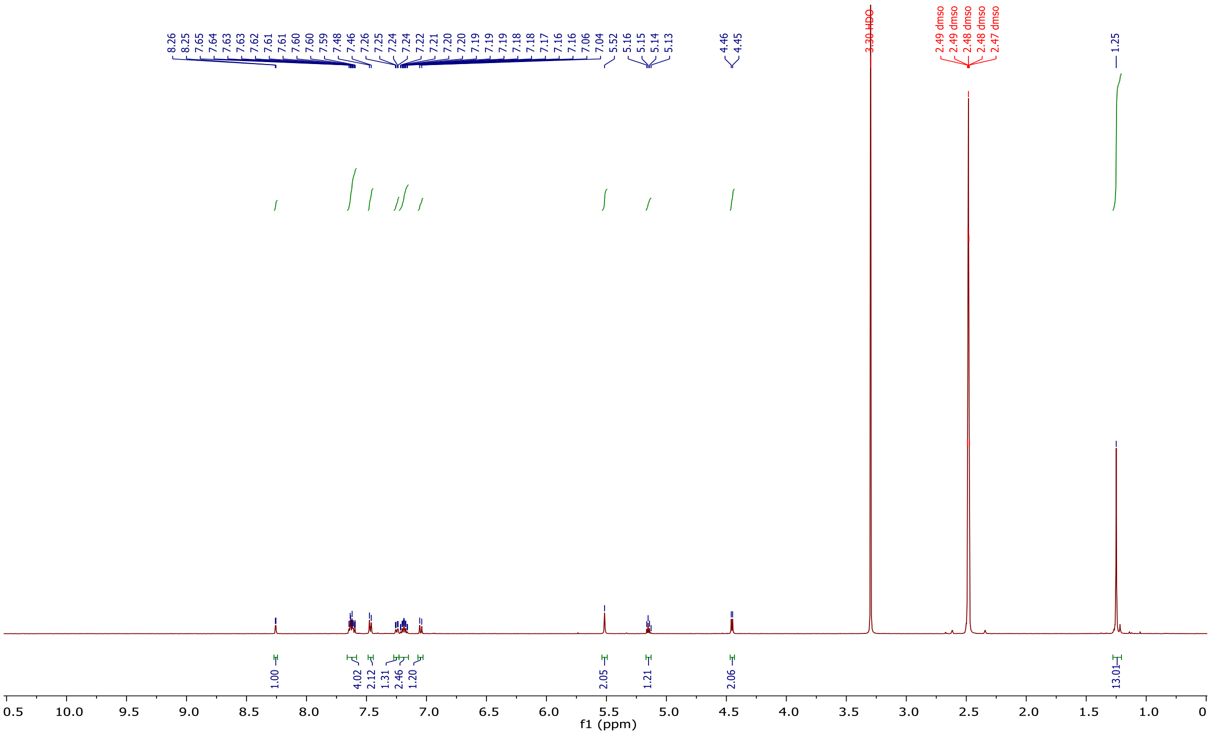

(3-(1*H*-benzo[d]imidazol-2-yl)-2-((4-(4,4,5,5-tetramethyl-1,3,2-dioxaborolan-2-yl)benzyl)oxy)phenyl)methanol (MO-E3) (126 MHz, DMSO-d_6)

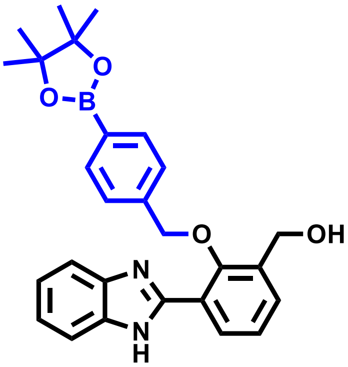

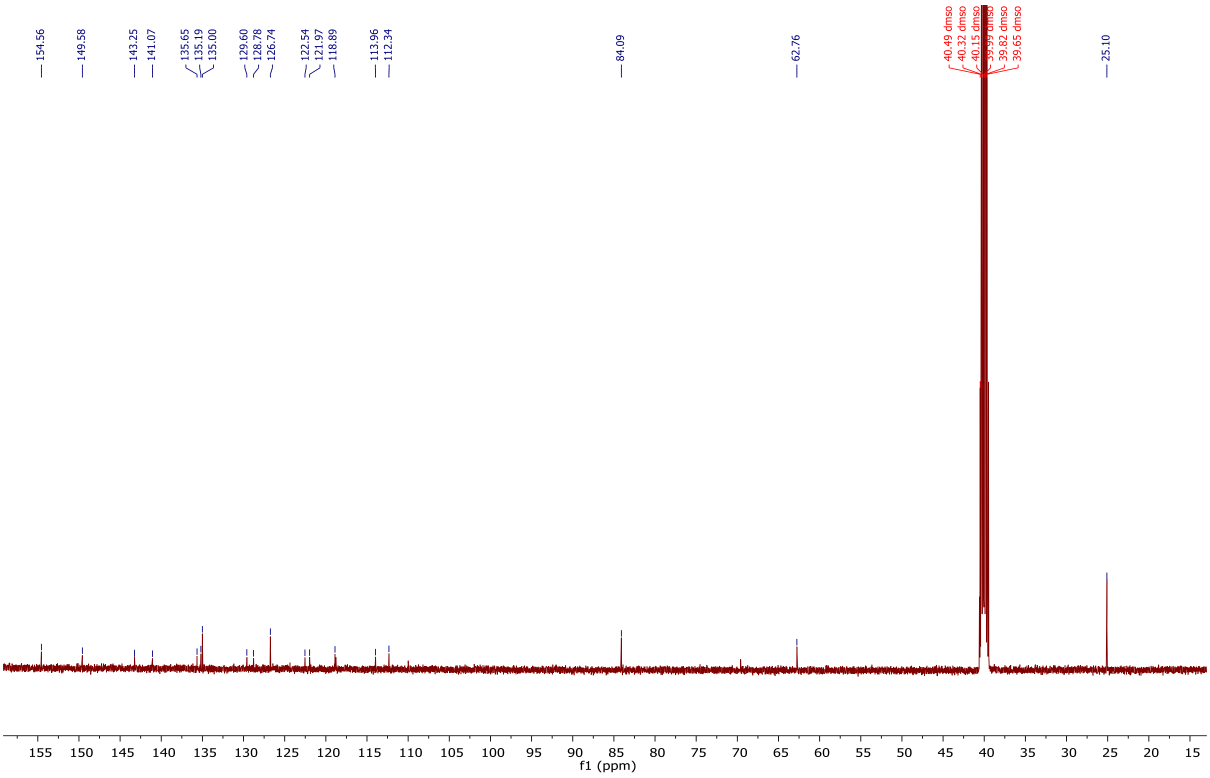

3-(1*H*-benzo[d]imidazol-2-yl)-2-((4-(4,4,5,5-tetramethyl-1,3,2-dioxaborolan-2-yl)benzyl)oxy)benzyl 2-(1-(4-chlorobenzoyl)-5-methoxy-2-methyl-1H-indol-3-yl)acetate (MO-E3) (500 MHz, $CDCl_3$)

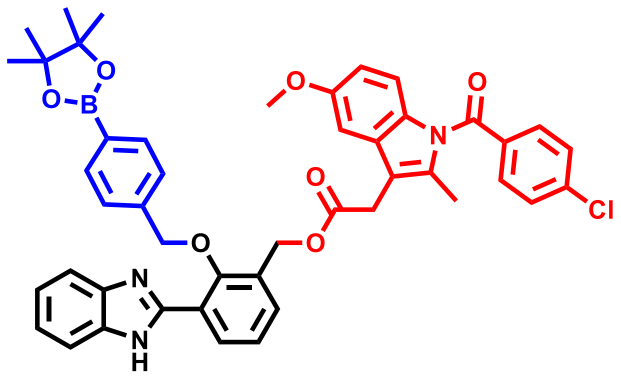

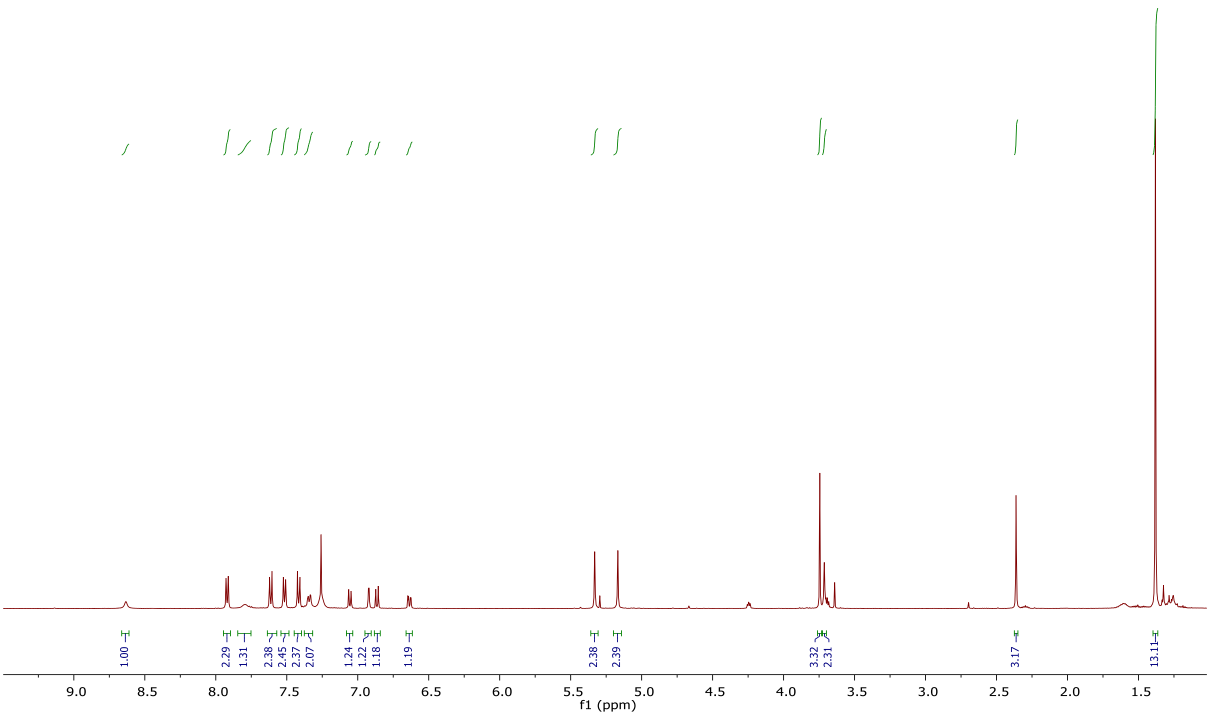

3-(1*H*-benzo[d]imidazol-2-yl)-2-((4-(4,4,5,5-tetramethyl-1,3,2-dioxaborolan-2-yl)benzyl)oxy)benzyl 2-(1-(4-chlorobenzoyl)-5-methoxy-2-methyl-1H-indol-3-yl)acetate (MO-E3) (126 MHz, $CDCl_3$)

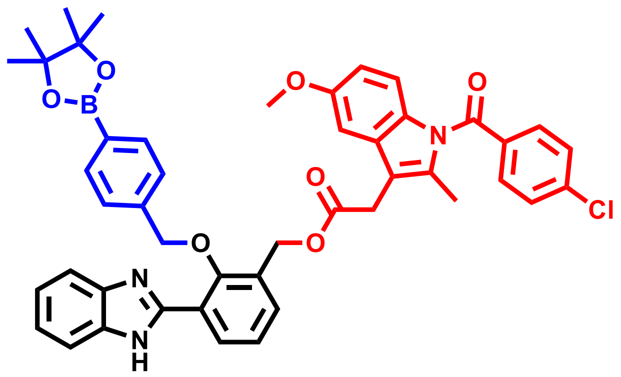

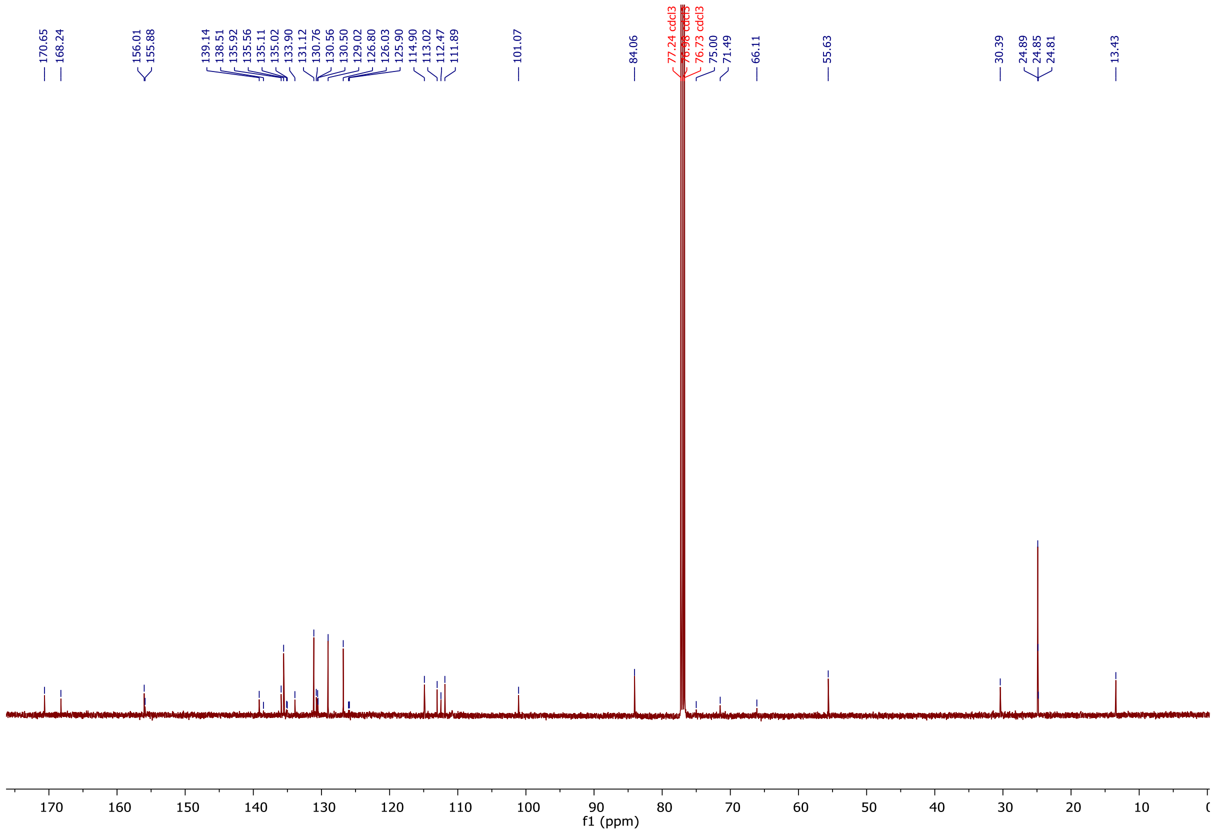

3. Preparation of ROS / RNS.

ROO·

ROO· was generated from 2,2'-azobis (2-amidinopropane) dihydrochloride. AAPH (2, 2′ azobis (2-amidinopropane) dihydrochloride,1 M) was added into deionizer water, and then stirred at 37 °C for 30 min.

$^{\cdot-}O_2$

Superoxide ($^{\cdot-}O_2$) was generated from KO_2. KO_2 and 18-crown-6 ether (2.5 eq) was dissolved in DMSO to afford a 0.25 M solution.

·OH

Hydroxyl radical (·OH) was generated by the Fenton reaction. To prepare •OH solution, hydrogen peroxide (H_2O_2, 10 eq) was added to $Fe(ClO_4)_2$ in deionised water.

$ONOO^-$

Simultaneously, 0.6 M KNO_2, 0.6 M HCl (aq) and 0.7 M in H_2O_2 in 10 mL H_2O were added to a 3 M NaOH solution (in 10 mL H_2O) at 0 ^{o}C. The concentration of $ONOO^-$ was determined by the absorption at 302 nm (Ɛ =1670 $cm^{-1}M^{-1}$) in 0.1 M aqueous sodium hydroxide solution.

^-OCl

The concentration of ^-OCl was determined from the absorption at 292 nm (Ɛ = 350 M^{-1} cm^{-1}).

H_2O_2

The concentration of H_2O_2 was determined from the absorption at 240 nm (Ɛ = 43.6 M^{-1} cm^{-1})

4. Materials and Methods

Measurement of Two-Photon Cross Section

The two-photon cross section (δ) was measured using Ti-sapphire femtosecond laser (Mai Tai HP). ESIPT probes (5.0×10^{-6} M) were dissolved in PBS buffer pH = 8.2 (52 % w/w H_2O: MeOH), and $ONOO^-$ was added (5.0×10^{-5} M). The two-photon induced fluorescence spectra was then obtained at 720−880 nm, and TPA was calculated using rhodamine 6G as the reference.

Two-Photon Microscopy Imaging

RAW264.7 macrophages were cultured in DMEM (WelGene) for two days. Before imaging, medium was changed to serum free medium and the chosen ESIPT probe (5 μM) was added. Images were acquired using multiphoton microscopes (Leica TCS SP8 MP) with Ti-sapphire femtosecond laser. Excitation wavelength was 740 nm and 2.49 W output power, which corresponded to 1.20×10^6 W cm^{-2} average power in the focal plane.

Cell viability.

CCK-8 kit (AbCareBio CL) assay was conducted to assess the cytotoxicity of each probe. RAW264.7 macrophages were cultured in 96-well plate for one day, and then difference concentrations of **MO-E2** (0–50 μM) were added. After incubation for 2 h, absorbances were measured at 450 nm.

Photostability

Photostability of **MO-E2** in RAW264.7 macrophages was conducted by monitoring the changes in fluorescence intensities, which was induced by two photon excitation. The fluorescence intensities were obtained 1800 signals with 2 sec intervals for 1 h. The fluorescence intensities were collected at 380–600 nm upon excitation at 740 nm.

Preparation and Staining of Fresh Rat Hippocampal Slices

A 14-day-old SD rats was used to perform tissue imaging. Rat hippocampal slices were cut into 0.4 mm thickness and transferred to glass-bottomed dishes (NEST). Slices were stained with **MO-E2** (50 μM) in DPBS (WelGene) and incubated for 1.5 h. Slices were washed with DPBS and observed using multiphoton microscopes.

5. UV-VIS analysis

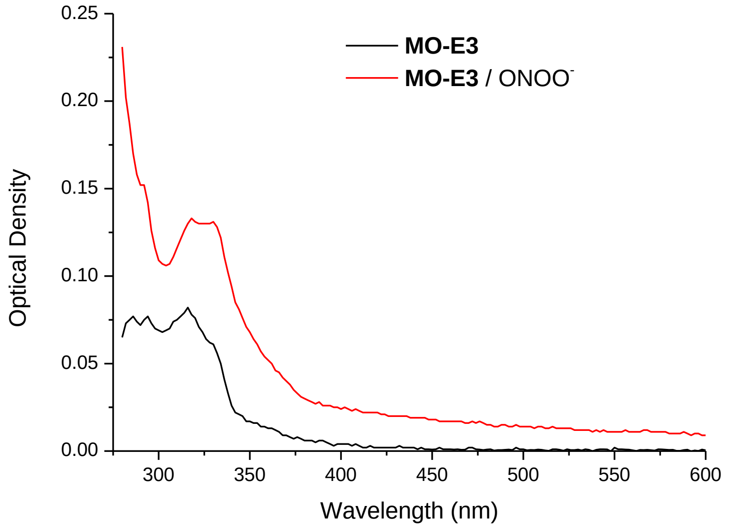

Figure S2- UV-Vis spectra of **MO-E3** (5 µM) with and without $ONOO^-$ (50 µM) in PBS buffer pH = 8.2 (52 % w/w H_2O: MeOH) at 25 ^oC.

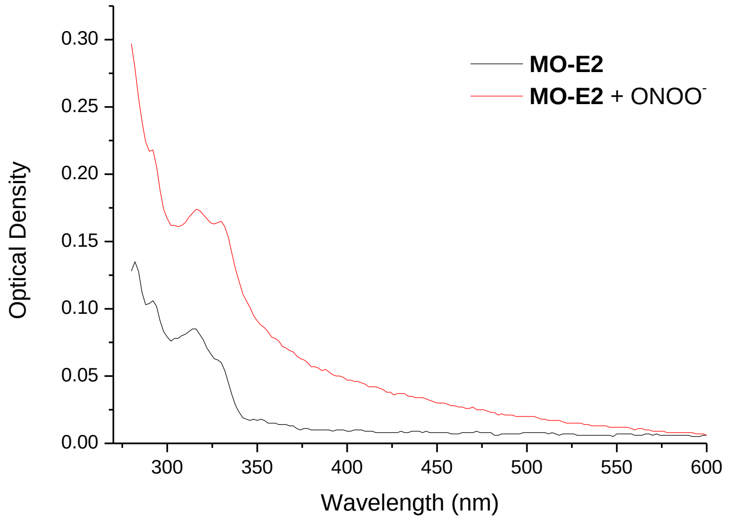

Figure S3 - UV-Vis spectra of **MO-E2** (5 µM) with and without $ONOO^-$ (50 µM) in PBS buffer pH = 8.2 (52 % w/w H_2O: MeOH) at 25 ^oC.

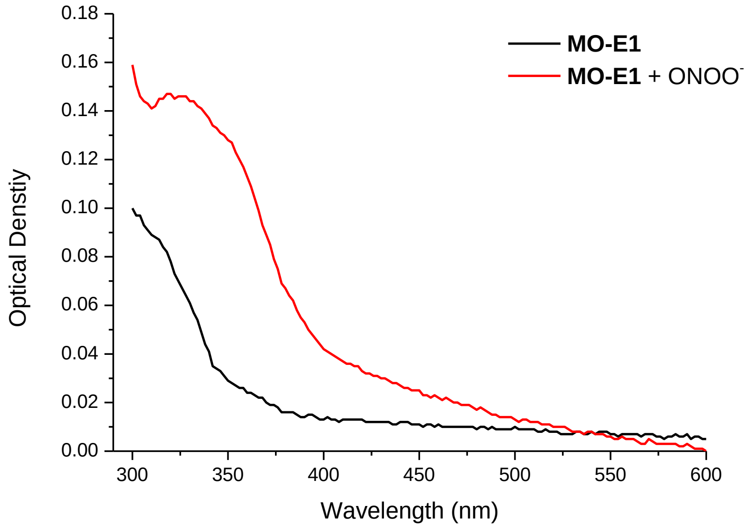

Figure S4- UV-VIS spectra of **MO-E1** (5 µM) with (red line) and without $ONOO^-$ (10 µM) in PBS buffer pH = 8.2 (52 % w/w H_2O:MeOH) at 25 ^oC.

6. Mechanism of activation

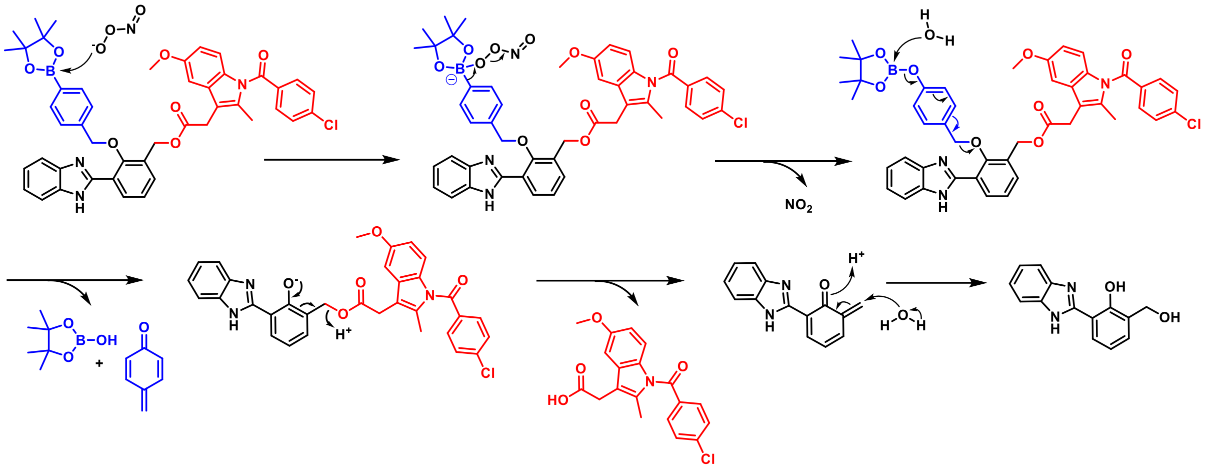

Figure S5 – Mechanism of activation of **MO-E3** on reaction with $ONOO^-$.

7. Mass spectrometry analysis.

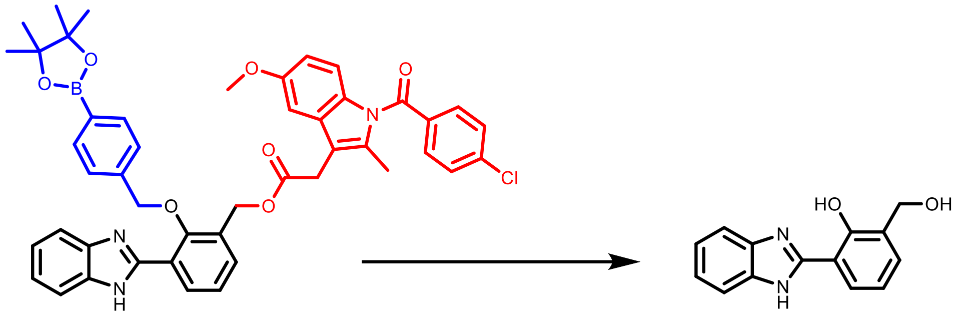

MO-E3

Molecular Formula: $C_{14}H_{12}N_2O_2$
Exact Mass: 240.0899
Observed Mass: 240.0882

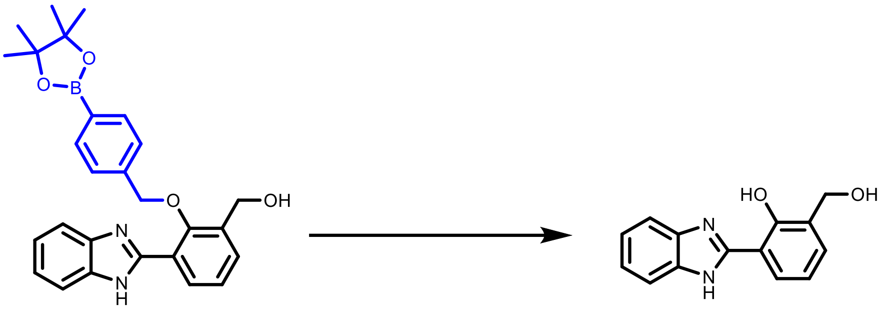

MO-E2

Molecular Formula: $C_{14}H_{12}N_2O_2$
Exact Mass: 240.0899
Observed Mass: 240.0897

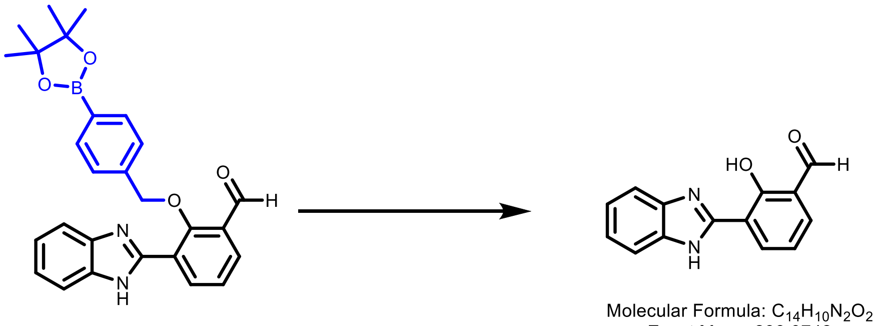

Molecular Formula: $C_{14}H_{10}N_2O_2$
Exact Mass: 238.0742
Observed Mass: 238.0739

MO-E1

Figure S6 – Mass spectrometry analysis of products of reaction of **MO-E1-3** with $ONOO^-$. The observed and exact mass are based on neutral mass analyses.

Walkup MS Report

UNIVERSITY OF BATH

Compound specific information

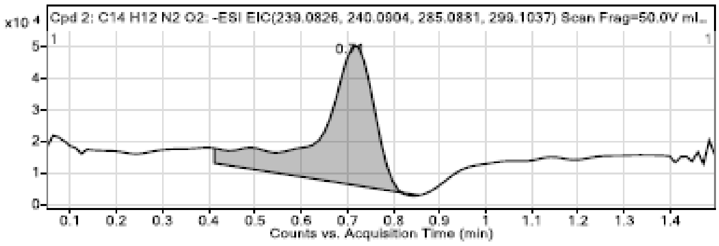

Figure: Extracted ion chromatogram (EIC) of compound.

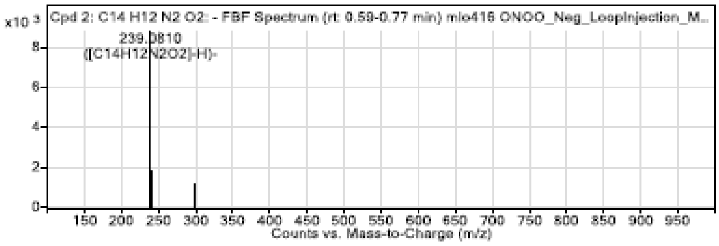

Figure: Full range view of Compound spectra and potential adducts.

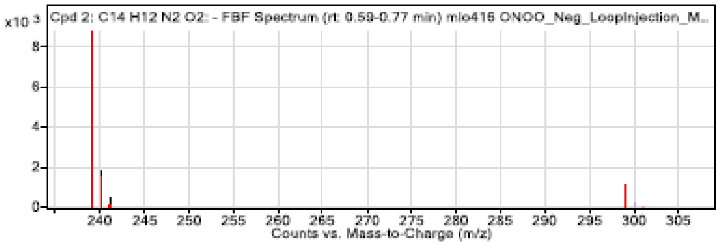

Figure: Zoomed Compound spectra view
(red boxes indicating expected theoretical isotope spacing and abundance)

Compound isotope peak List

m/z	z	Abund	Formula	Ion
239.0810	1	8816.7	C14H12N2O2	(M-H)-
240.0855	1	1832.5	C14H12N2O2	(M-H)-
241.0869	1	500.3	C14H12N2O2	(M-H)-
299.0988	1	1137.0	C14H12N2O2	(M+CH3COO)-

Figure S7 – Mass Spectroscopic data of **MO-E3** in the presence of ONOO$^-$

Walkup MS Report

Compound specific information

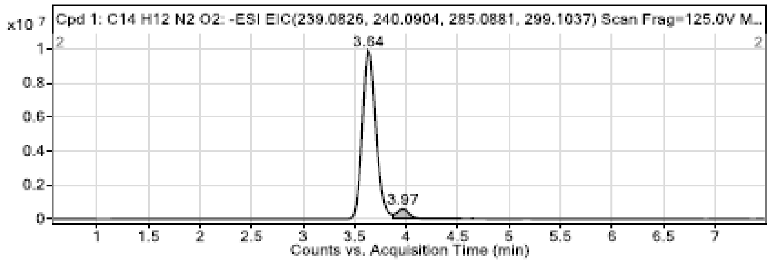

Figure: Extracted ion chromatogram (EIC) of compound.

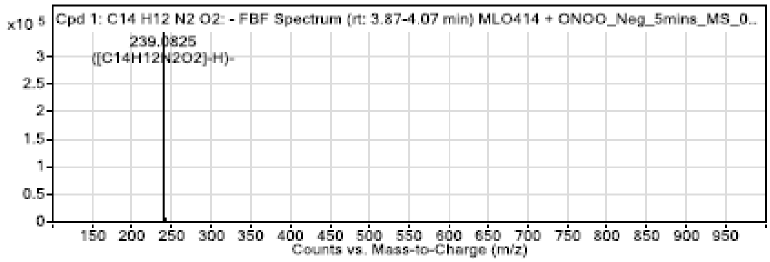

Figure: Full range view of Compound spectra and potential adducts.

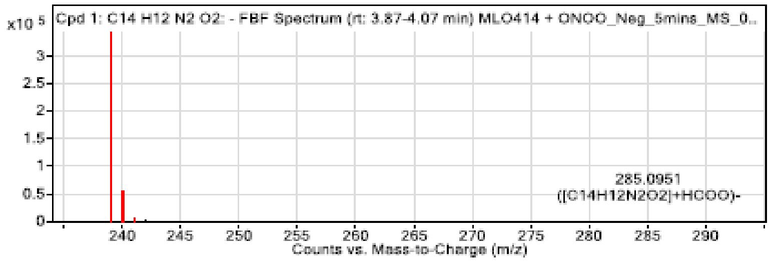

Figure: Zoomed Compound spectra view
(red boxes indicating expected theoretical isotope spacing and abundance)

Compound isotope peak List

m/z	z	Abund	Formula	Ion
239.0825	1	341418.3	C14H12N2O2	(M-H)-
240.0855	1	52631.9	C14H12N2O2	(M-H)-
241.0875	1	5430.3	C14H12N2O2	(M-H)-
242.0619	1	956.9	C14H12N2O2	(M-H)-
243.0653	1	474.3	C14H12N2O2	(M-H)-
285.0951	1	295.9	C14H12N2O2	(M+HCOO)-
286.1207	1	201.8	C14H12N2O2	(M+HCOO)-

--- End Of Report ---

Figure S8 - Mass Spectroscopic data of **MO-E2** in the presence of $ONOO^-$

Walkup MS Report

Compound specific information

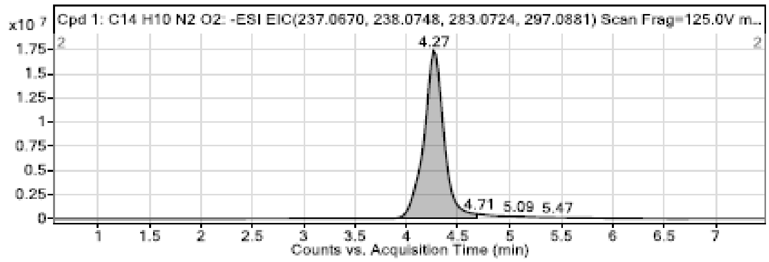

Figure: Extracted ion chromatogram (EIC) of compound.

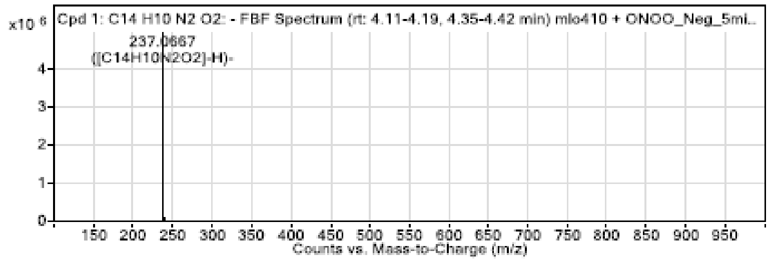

Figure: Full range view of Compound spectra and potential adducts.

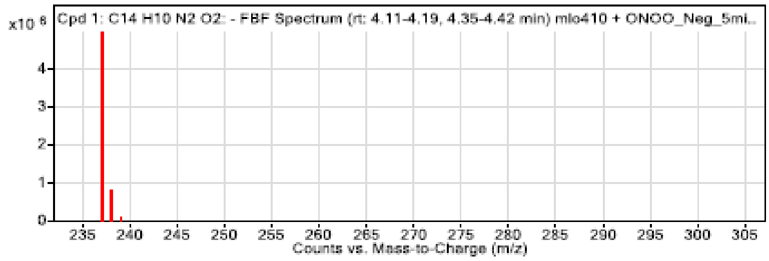

Figure: Zoomed Compound spectra view
(red boxes indicating expected theoretical isotope spacing and abundance)

Compound isotope peak List

m/z	z	Abund	Formula	Ion
237.0667	1	4927698.0	C14H10N2O2	(M-H)-
238.0698	1	727077.2	C14H10N2O2	(M-H)-
239.0724	1	68637.7	C14H10N2O2	(M-H)-
240.0723	1	6467.6	C14H10N2O2	(M-H)-
297.0870	1	3880.3	C14H10N2O2	(M+CH3COO)-
298.0457	1	6306.6	C14H10N2O2	(M+CH3COO)-
299.0328	1	1022.0	C14H10N2O2	(M+CH3COO)-

--- End Of Report ---

Figure S9 -Mass Spectroscopic data of **MO-E1** in the presence of $ONOO^-$

LC-MS analysis

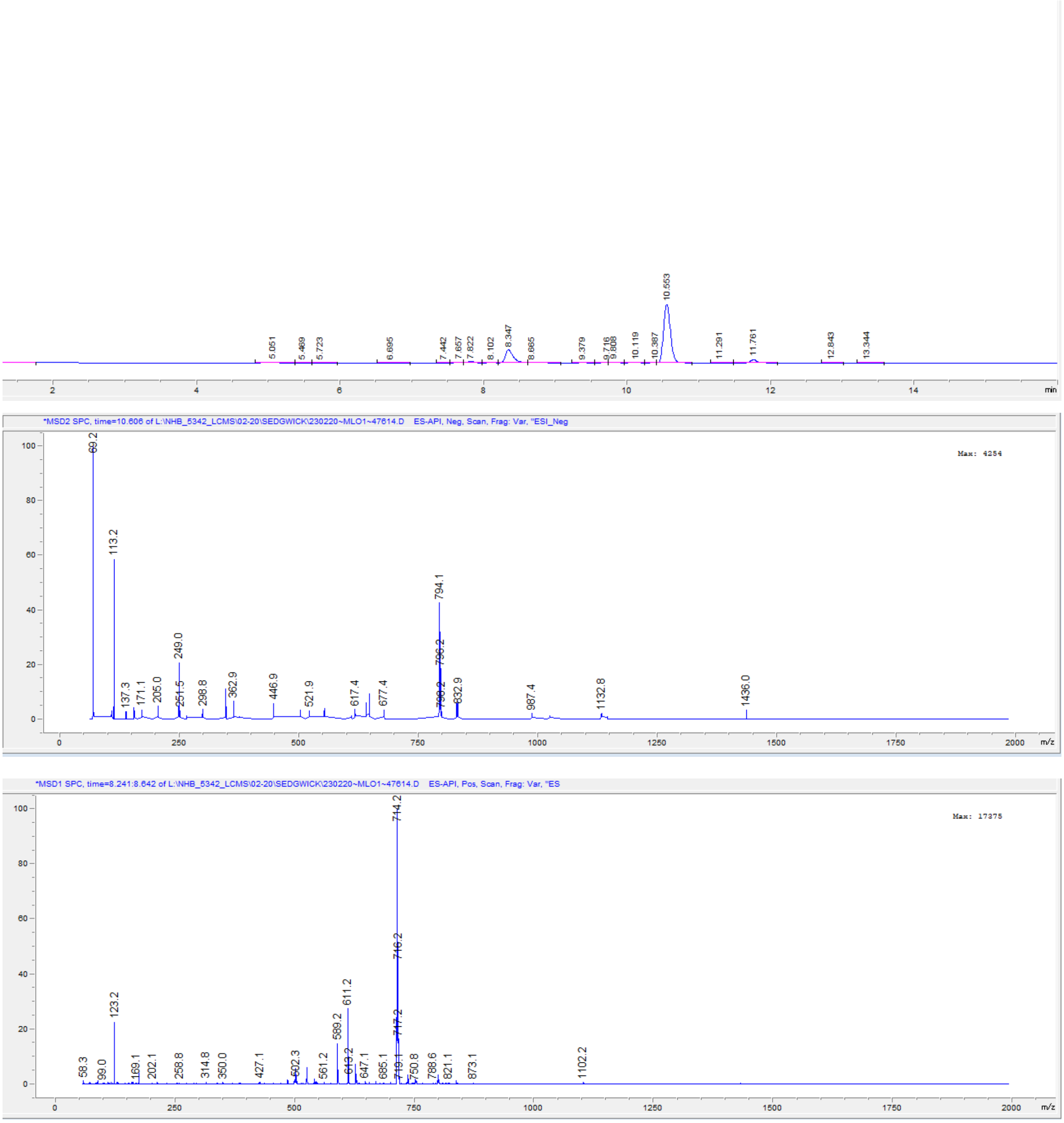

Figure S10 – LCMS data of **MO-E3** (MeCN/$H_2$0; 5:95 5 min hold to 100 % linear gradient). Top: UV chromatogram. Middle: MS chromatogram representative of peak at t = 10.606 min; determining **MO-E3**- ($C_{46}H_{43}O_7N_3BCl$) with m/z 794.2 ($[M+H]^+$). Bottom: MS chromatogram representative of peak at t = 8.241; determining boronic acid (loss of pinacol - $C_{40}H_{33}O_7N_3BCl$) with 714.2 ($[M+H]^+$)

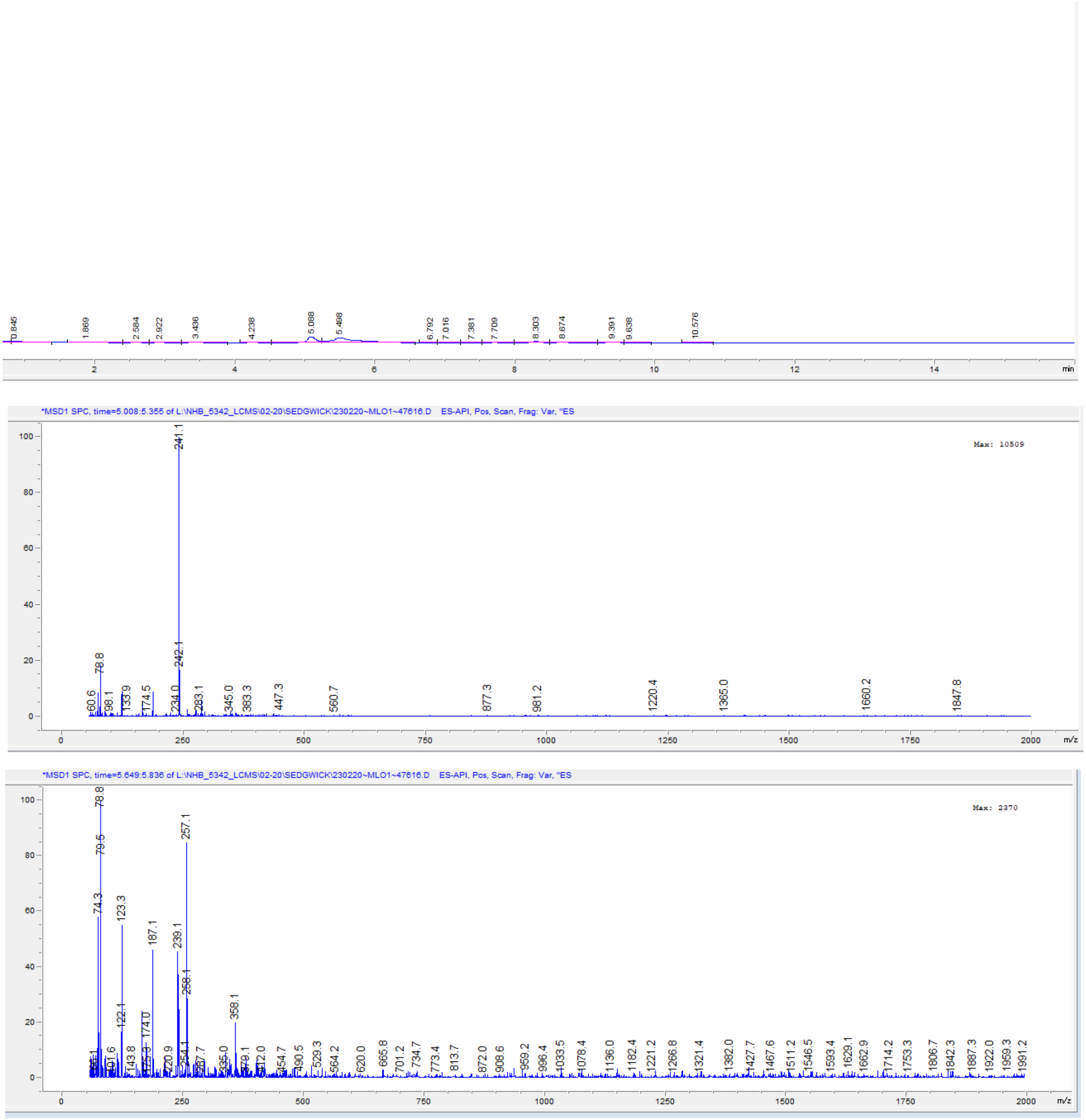

Figure S11 - LCMS data of **MO-E3** in the presence of H_2O_2 (MeCN/$H_2$0; 5:95 5 min hold to 100 % linear gradient). Top: UV chromatogram. Middle: MS chromatogram representative of peak at t = 5.088 min; fragments determining fluorescent product ($C_{14}H_{12}N_2O_2$) found 241.1 ($[M+H]^+$). Bottom: MS chromatogram representative of peak at t = 5.649; determining indomethacin ($C_{19}H_{16}ClNO_4$) found 358.1 for ($[M+H]^+$).

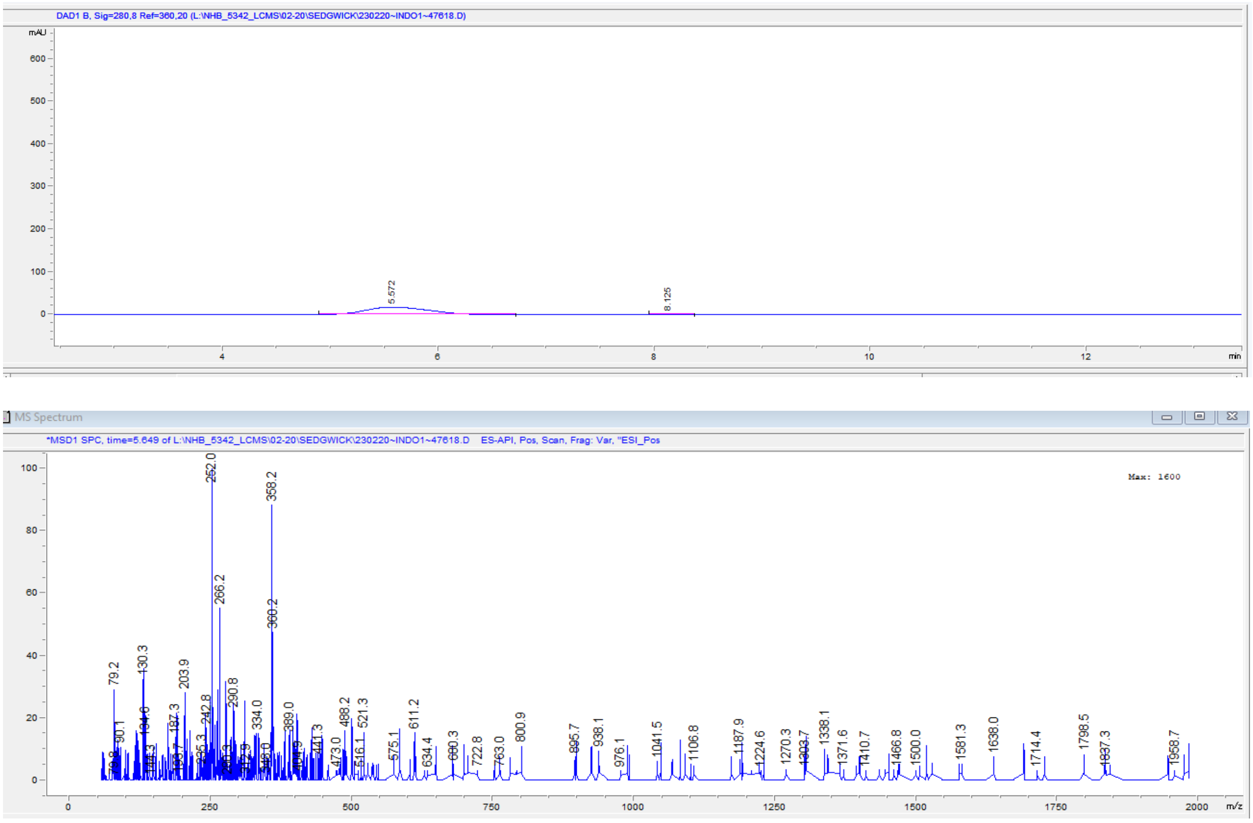

Figure S12 - LCMS data of **Indomethacin** (MeCN/H_20; 5:95 5 min hold to 100 % linear gradient).Top: UV chromatogram. Bottom: MS chromatogram representative of peak at t = 5.649; determining indomethacin ($C_{19}H_{16}ClNO_4$) found 358.1 for ($[M+H]^+$).

8. Fluorescence Analysis

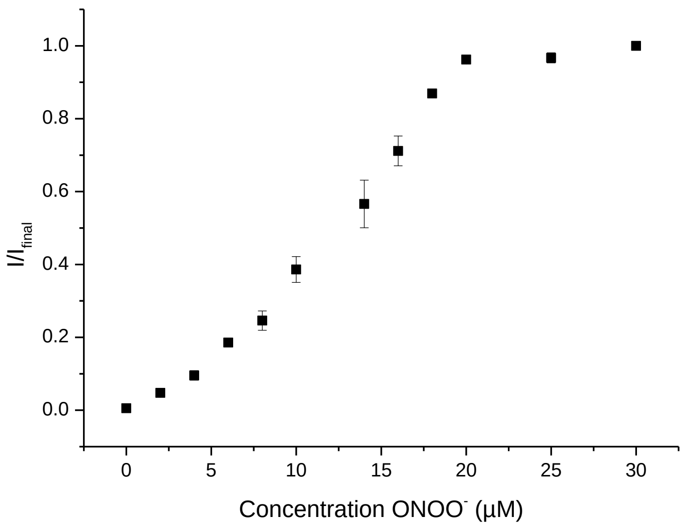

Figure S13 - Relative fluorescence intensity of **MO-E3** (5 µM) at 450 nm in the presence of $ONOO^-$ (0-30 µM) in PBS buffer pH = 8.2 (52 % w/w H_2O: MeOH) at 25 ºC, λ_{ex} = 325 (bandwidth: 16) nm on a BMG Labtech CLARIOstar® plate reader.

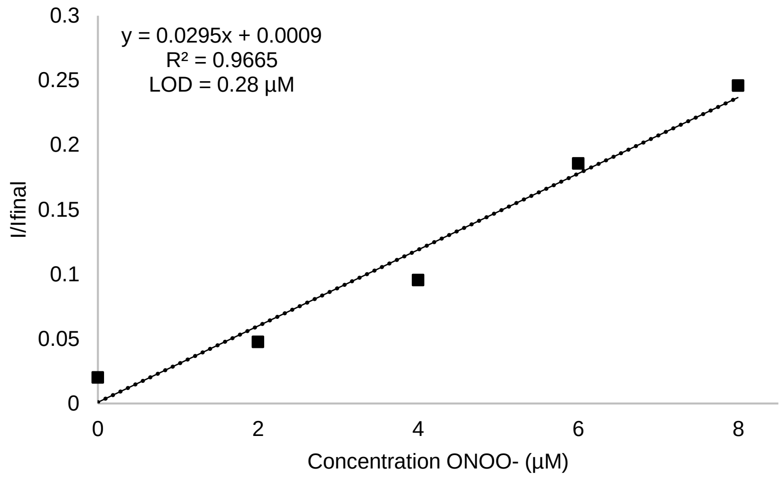

Figure S14 - Plots of relative fluorescence intensity of **MO-E3** (5 µM) as a function of $ONOO^-$ concentration at λ_{max}= 450 nm. Fluorescence studies were carried out in PBS buffer pH = 8.2 (52 % w/w H_2O : MeOH) at 25 ºC, λ_{ex}= 325 (bandwidth: 16) nm on a BMG Labtech CLARIOstar® plate reader. Calculation for LOD = limit of detection (3σ/k) – of the linear section of the graph.

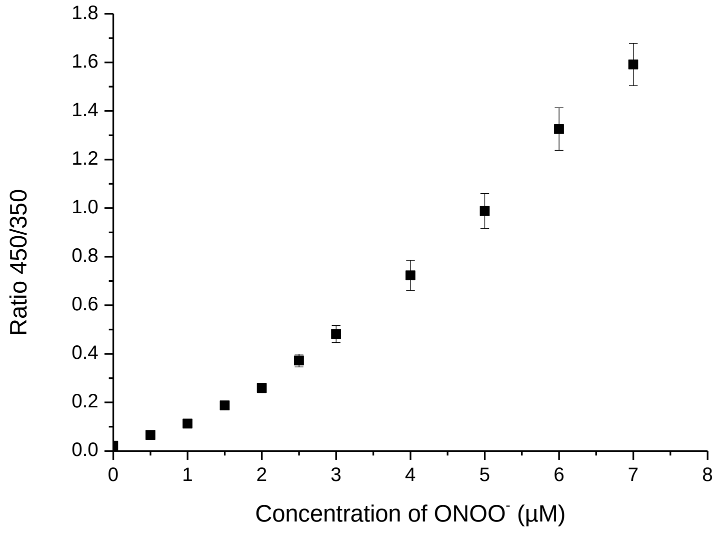

Figure S15 - Relative ratio of **MO-E2** (5 µM) at 450 nm and 350 nm in the presence of $ONOO^-$ (0-7 µM) in PBS buffer pH = 8.2 (52 % w/w H_2O: MeOH) at 25 ^oC, λ_{ex} = 325 (bandwidth: 16) nm on a BMG Labtech CLARIOstar® plate reader.

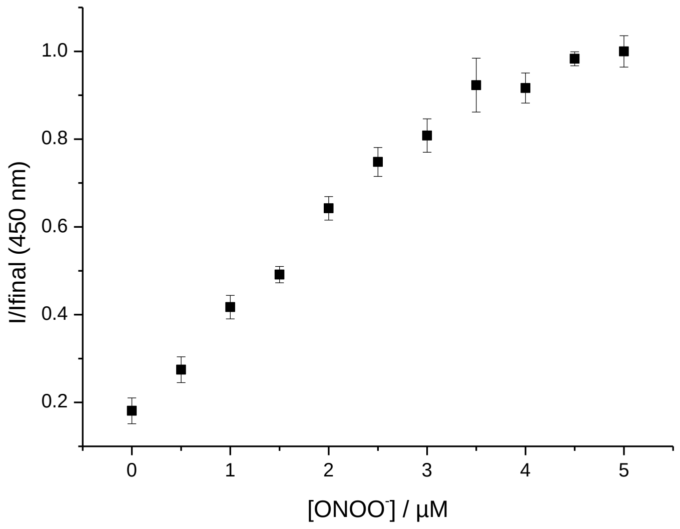

Figure S16 - Dose dependence curve of **MO-E2** (5 µM) in the presence of $ONOO^-$ (0 - 5 µM) in PBS buffer pH = 8.2 (52 % w/w H_2O : MeOH) at 25 ^oC, $\lambda_{ex=}$ = 325 (bandwidth: 16) nm on a BMG Labtech CLARIOstar® plate reader, λ_{max}= 450 nm.

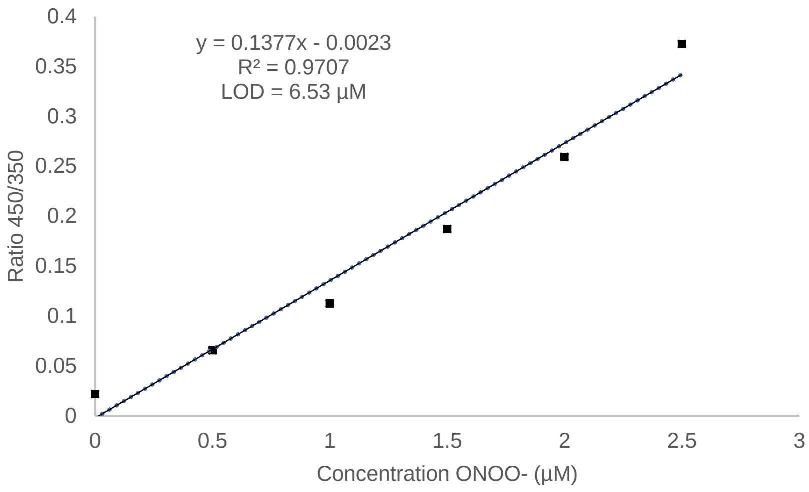

Figure S17 - Plots of relative fluorescence intensity of **MO-E2** (5 µM) as a function of $ONOO^-$ concentration at λ_{max}= 450/350 nm. Fluorescence studies were carried out in PBS buffer pH = 8.2 (52 % w/w H_2O: MeOH) at 25 ^oC, λ_{ex}=325 (bandwidth: 16) nm on a BMG Labtech CLARIOstar® plate reader. Calculation for LOD = limit of detection (3σ/k) – of the linear section of the graph.

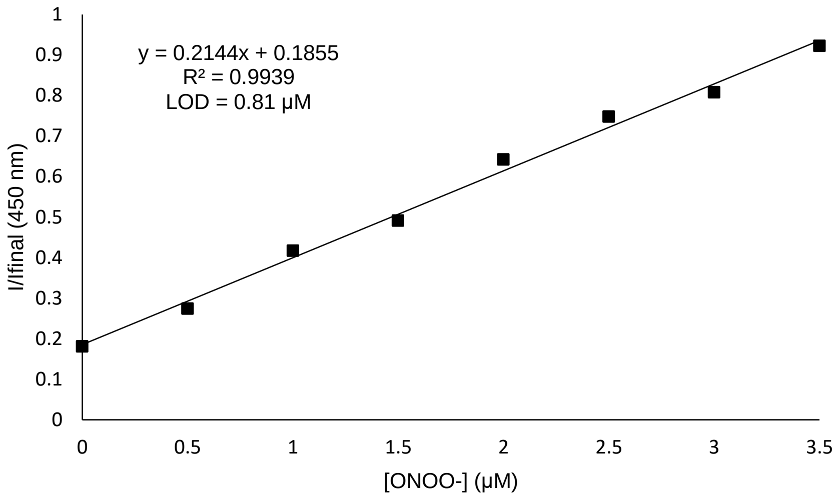

Figure S18 - Plots of relative fluorescence intensity of **MO-E1** (5 µM) as a function of $ONOO^-$ concentration λ_{max}= 450 nm. Fluorescence studies were carried out in PBS buffer pH = 8.2 (52 % w/w H_2O: MeOH) at 25 ^oC, λ_{ex}= 325 (bandwidth: 16) nm on a BMG Labtech CLARIOstar® plate reader. Calculation for LOD = limit of detection (3σ/k) – of the linear section of the graph.

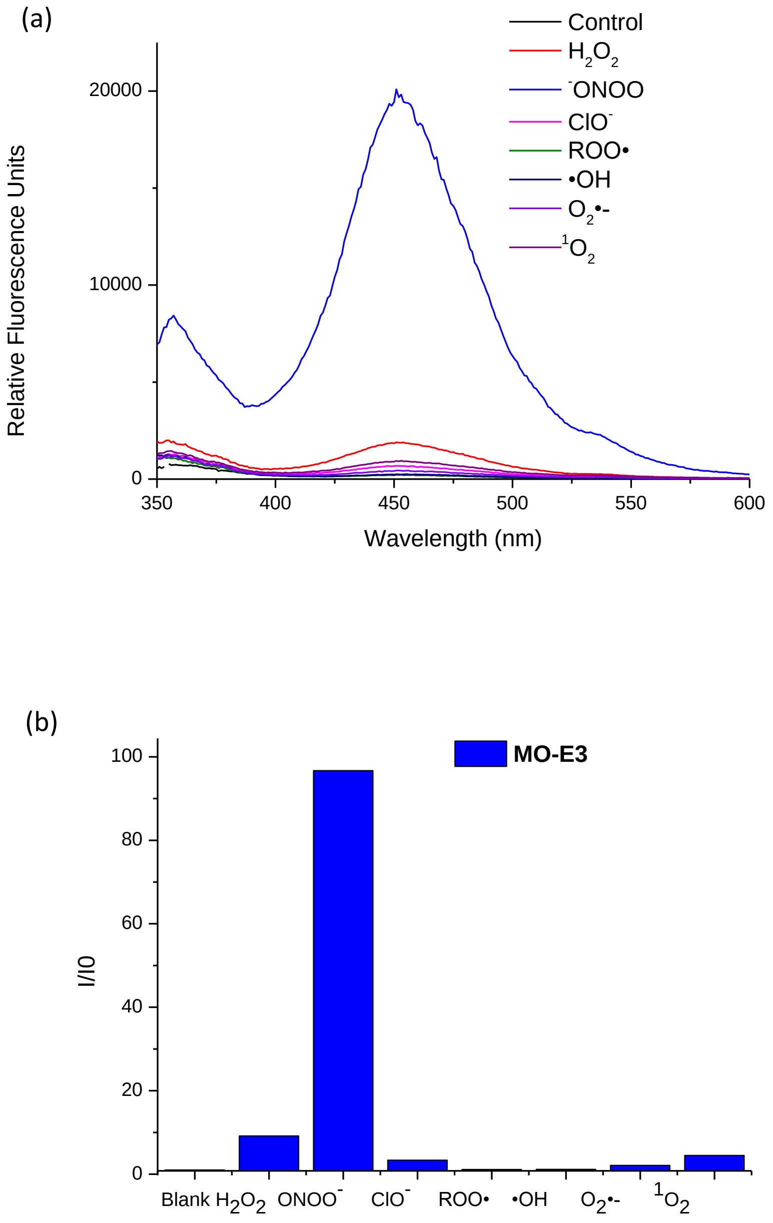

Figure S19- (a) Fluorescence spectra of **MO-E3** (5 µM) in the presence of $ONOO^-$ (50 µM), OH (100 µM), $O_2^{\cdot-}$ (100 µM), $^1O_2$ (100 µM), ClO^- (100 µM), ROO· (100 µM) and H_2O_2 (100 µM). (b) Fluorescence selectivity of **MO-E3** (5 µM) in the presence of $ONOO^-$ (10 µM), ·OH (100 µM), $O_2^{\cdot-}$ (100 µM), $^1O_2$ (100 µM), ClO^- (100 µM), ROO· (100 µM) and H_2O_2 (100 µM). The fluorescence intensity was taken at λ_{max}= 450 nm. All fluorescence measurements were taken after 30 mins incubation, in PBS buffer pH = 8.2 (52 % w/w H_2O: MeOH) at 25 °C, λ_{ex} = 325 (bandwidth: 16) nm on a BMG Labtech CLARIOstar® plate reader.

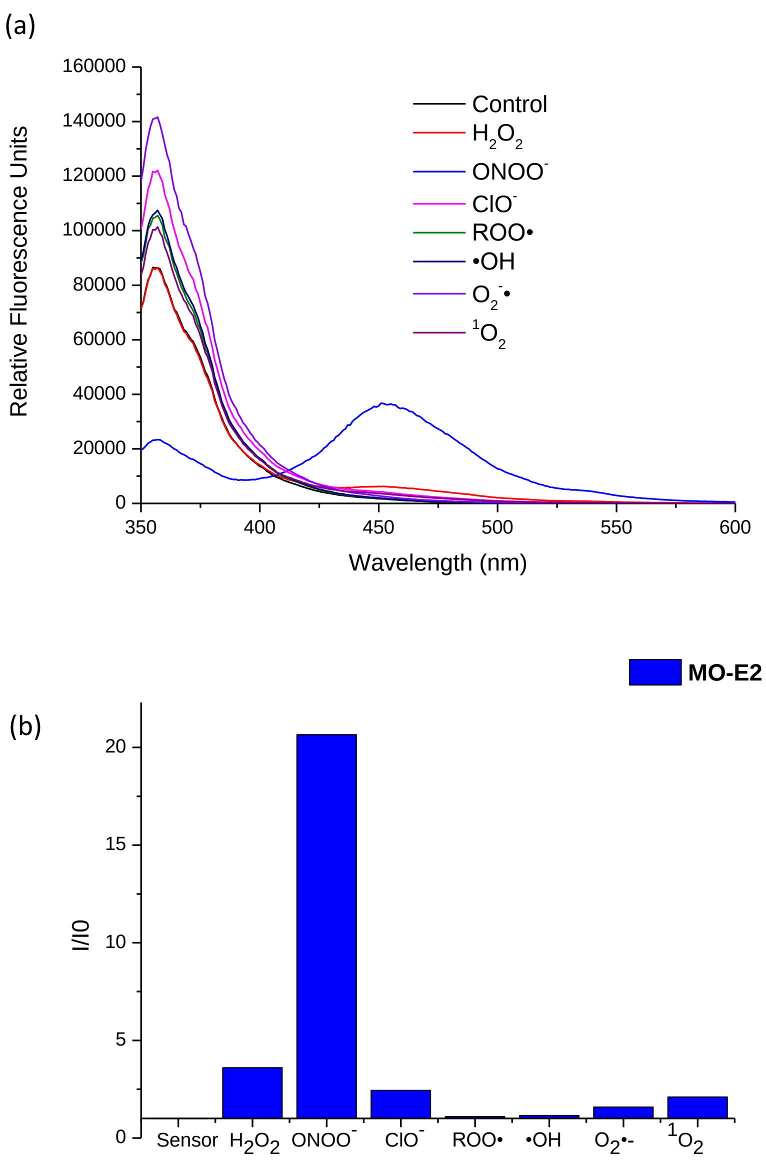

Figure S20- (a) Fluorescence spectra of **MO-E2** (5 µM) in the presence of $ONOO^-$ (50 µM), ·OH (100 µM), $O_2^{\cdot -}$ (100 µM), $^1O_2$ (100 µM), ClO^- (100 µM), ROO· (100 µM) and H_2O_2 (100 µM). (b) Fluorescence selectivity of **MO-E2** (5 µM) in the presence of $ONOO^-$ (10 µM), ·OH (100 µM), $O_2^{\cdot -}$ (100 µM), $^1O_2$ (100 µM), ClO^- (100 µM), ROO· (100 µM) and H_2O_2 (100 µM). The fluorescence intensity was taken at λ_{max}= 450 nm. All fluorescence measurements were taken after 30 mins incubation, in PBS buffer pH = 8.2 (52 % w/w H_2O: MeOH) at 25 °C, λ_{ex} = 325 (bandwidth: 16) nm on a BMG Labtech CLARIOstar® plate reader.

(a)

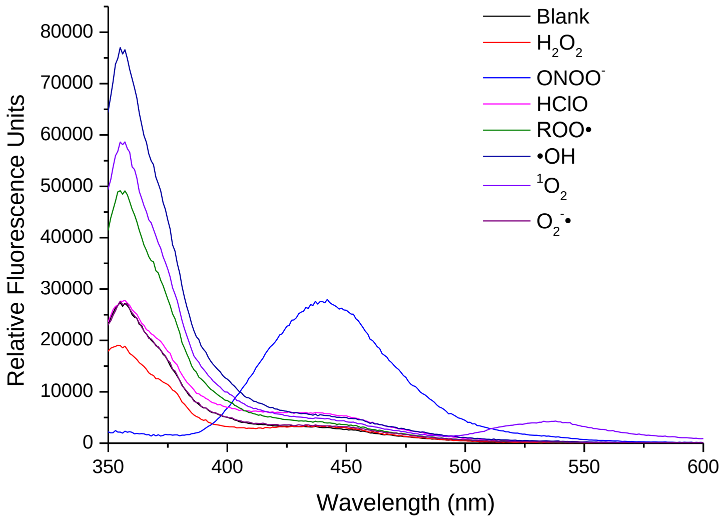

(b)

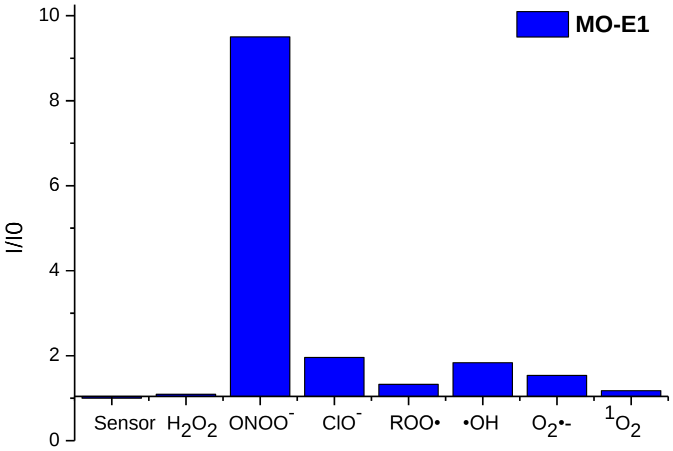

Figure S21- (a) Fluorescence spectra of **MO-E1** (5 µM) in the presence of $ONOO^-$ (10 µM), OH (100 µM), $O_2^{\cdot-}$ (100 µM), $^1O_2$ (100 µM), ClO^- (100 µM), ROO· (100 µM) and H_2O_2 (100 µM). (b) Fluorescence selectivity of **MO-E1** (5 µM) in the presence of $ONOO^-$ (10 µM), OH (100 µM), $O_2^{\cdot-}$ (100 µM), $^1O_2$ (100 µM), ClO^- (100 µM), ROO· (100 µM) and H_2O_2 (100 µM). The fluorescence intensity was taken at λ_{max}= 450 nm. All fluorescence measurements were taken after 30 mins incubation, in PBS buffer pH = 8.2 (52 % w/w H_2O: MeOH) at 25 ºC, λ_{ex} = 325 (bandwidth: 16) nm on a BMG Labtech CLARIOstar® plate reader.

9. Cell studies.

Table S1 – Photopysical data for ESIPT probes.

Compound	$\lambda^{(2)}_{max}$ [a]	$\Phi\delta_{max}$ [b]
**MO-E3**	740	0.25
**MO-E3** + $ONOO^-$	740	0.67
**MO-E2**	740	0.28
**MO-E2** + $ONOO^-$	740	1.24
**MO-E1**	740	0.26
**MO-E1** + $ONOO^-$	740	0.27

a) λ_{max} of the two-photon emission spectra in nm. b) Φ is the fluorescence quantum yield and δ_{max} is the two-photon action cross sections in GM (1 GM = 10^{-50} cm^4 s $photon^{-1}$).

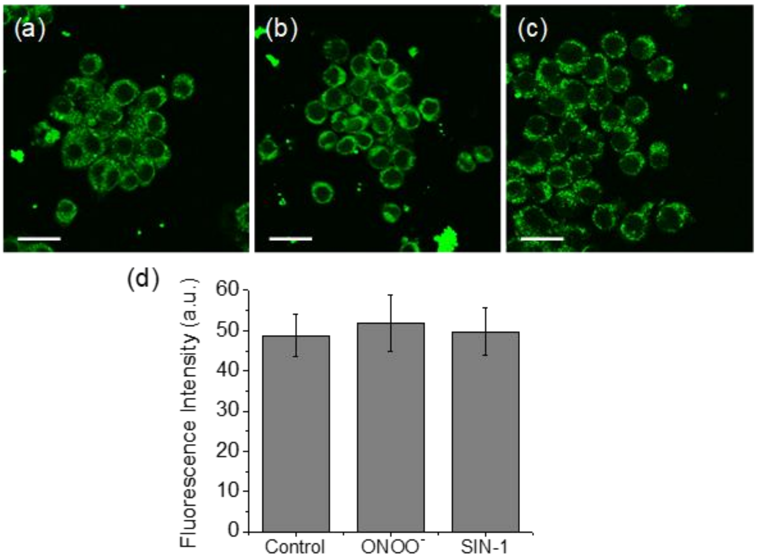

Figure S22 - TPM fluoresce images of RAW264.7 macrophages labelled with **MO-E3** (5 µM) for 30 min. (a) Control image. (b–c) Cells were pre-treated with (b) exogenous $ONOO^-$ (50 µM, 30 min), (c) SIN-1 (50 µM, 30 min) (d) Average fluorescence intensity in the corresponding TPM fluorescence images. Excitation wavelength and detection windows were 740 nm and 380–600 nm, respectively. Scale bars = 20 µm.

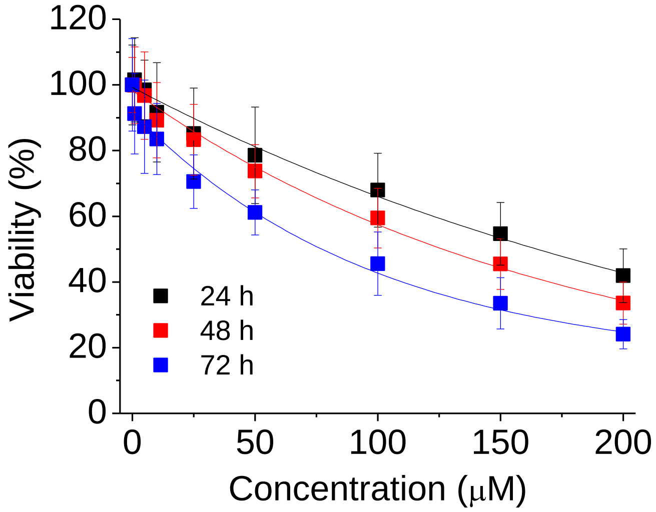

Figure S23 - Cytotoxicity assays of **MO-E2** labelled RAW264.7 macrophages using CCK-8 to determine the IC50. Cells were incubated with 0–200 μM of **MO-E2** and the concentration of **MO-E2** which exhibited 50 % cell viability for 24, 48, and 72 h were 163.6, 125.3 and 74.7 μM, respectively.
